# YWHAG inhibits influenza a virus replication by suppressing the release of viral M2 protein

**DOI:** 10.3389/fmicb.2022.951009

**Published:** 2022-07-19

**Authors:** Haiying Mao, Lei Cao, Ting Xu, Xiaohan Xia, Peilei Ren, Pengfei Han, Chengfei Li, Xianfeng Hui, Xian Lin, Kun Huang, Meilin Jin

**Affiliations:** ^1^State Key Laboratory of Agricultural Microbiology, Huazhong Agricultural University, Wuhan, China; ^2^College of Veterinary Medicine, Huazhong Agricultural University, Wuhan, China; ^3^Key Laboratory of Development of Veterinary Diagnostic Products, Ministry of Agriculture, Wuhan, China; ^4^State Key Laboratory of Biocatalysis and Enzyme Engineering, School of Life Sciences, Hubei University, Wuhan, China; ^5^College of Science, Huazhong Agricultural University, Wuhan, China; ^6^Chinese Academy of Sciences (CAS) Key Laboratory of Special Pathogens and Biosafety, Wuhan Institute of Virology, Wuhan, China

**Keywords:** influenza A virus, YWHAG, M2 protein, viral budding, protein–protein interaction

## Abstract

Influenza A virus (IAV) poses a serious threat to human life and property. The IAV matrix protein 2 (M2) is significant in viral budding. Increasing studies have proven the important roles of host factors in IAV replication. In this study, immunoprecipitation combined with mass spectrometry revealed that the host protein *tyrosine 3-monooxygenase/tryptophan 5-monooxygenase activation protein gamma* (YWHAG), which belongs to the 14-3-3 protein scaffold family, interacts with M2. Their interactions were further confirmed by co-immunoprecipitation (Co-IP), immunofluorescence, and confocal microscopy of virus-infected HeLa cells. Moreover, we constructed YWHAG-KO and YWHAG-overexpressing cells and found that YWHAG knockout significantly increased viral production, whereas its overexpression reduced the titer of virus progeny. Therefore, YWHAG is a negative regulatory factor during IAV infection. Further, YWHAG knockout or overexpression had no effect on the binding, entry, or viral RNA replication in the early stages of the virus life cycle. On the contrary, it impaired the release of virions at the plasma membrane as determined using transmission electron microscopy and suppressed the M2-mediated budding of the influenza virus. Importantly, the H158F mutation of YWHAG was found to affect interaction with M2 and its budding. Collectively, our work demonstrates that YWHAG is a novel cellular regulator that targets and mediates the interaction and release of M2.

## Introduction

Influenza is an important cause of human and animal morbidity and has long been the focus of national concern (Chawla et al., 2009). Influenza A virus (IAV) is a segmented negative-stranded RNA virus that contains eight segments encoding approximately 14 proteins, polymerase protein (PB1, PB2, and PA), nucleoprotein (NP), hemagglutinin (HA) and neuraminidase (NA), Matrix protein 1 (M1) and Matrix protein 2 (M2), non-structural protein (NS1 and NS2) (Wang et al., 2019; Lutz Iv et al., 2020; Borkenhagen et al., 2021; Noda, 2021; Pinto et al., 2021). Each protein has its own function in the IAV. Similarly, the M2 protein plays important roles in the viral life cycle (Chauhan and Gordon, 2022). M2 contains 97 amino acid residues and assembles into a homotetramer to constitute three different domains that include a 24-residue N-terminal ectodomain (ED), a single 19-residue transmembrane domain (TM) that forms the pore, and a long 54-residue carboxyterminal cytoplasmic domain called cytoplasmic tail (CT) (Mezhenskaya et al., 2019; Manzoor et al., 2020; Okuya et al., 2020; Movellan et al., 2021), which is highly conserved among viral strains (Wohlgemuth et al., 2018; Kim et al., 2019).

TM of M2 forms the ion channel and M2 possesses a proton-selective ion channel activity, which was created by the low pH environment of the endosome (Manzoor et al., 2017). M2 allows fusion of the virus envelope and endosomal membrane, leading to the release of viral ribonucleoproteins (vRNPs) (Lin et al., 2017; Claridge et al., 2020; Lamb, 2020; Torabifard et al., 2020). Besides, M2 contributes to viral assembly and budding (Paulino et al., 2019). Recent studies have indicated that the M2 CT amphipathic helix modifies the membrane curvature at the neck of the budding virus in a cholesterol-dependent manner, causing membrane scission and completion of the budding process with the release of the progeny virion (Elkins et al., 2017; Martyna et al., 2020).

An increasing number of studies have examined host proteins that regulate the life cycle of influenza virus by interacting with the viral M2 protein. For example, M2 forms a stable complex with Hsp40 and P58, which enhances PKR phosphorylation and induces cell death, thereby affecting influenza virus replication (Goodman et al., 2007; Guan et al., 2010). The host protein MARCH8 can target viral M2 in a ubiquitination-dependent manner and inhibit virus proliferation (Liu et al., 2021b). Further, M2 protein degrades BST-2 protein through the proteasome pathway and reduces the possibility of IAV production (Hu et al., 2017). The host protein TRAPPC6Aδ interacts with M2 and blocks its transport to the apical plasma membrane, which is beneficial for virus replication *in vitro* and regulates virulence in mice (Zhu et al., 2017).

The 14-3-3 protein family includes seven isoforms that are highly conserved in sequence and structure (Zhang et al., 2021). The 14-3-3 proteins are involved in almost all the important cellular processes, such as transcription, regulation of cell cycle and metabolism, intracellular transport, cytoskeletal structure, and apoptosis (Fu et al., 2000; Obsil and Obsilova, 2011; Obsilova et al., 2014; Aghazadeh and Papadopoulos, 2016). Most of them regulate the conformation of target proteins, the localization of target proteins in cells, and act as anchor proteins (Sluchanko and Gusev, 2010). Some studies have examined the function of 14-3-3 proteins in viral infection; for instance, 14-3-3ε has been reported to competitively bind RIG-I with the NS3 protein of Zika virus, resulting in blocked binding of RIG-I with downstream MAVS and weakening the antiviral effect mediated by RIG-I (Riedl et al., 2019). Interaction of the host protein 14-3-3 with the parainfluenza virus 5M protein negatively affects virus particle formation (Pei et al., 2011). In addition to being associated with RNA viruses, 14-3-3 proteins play a vital role in DNA viruses through RLR signaling pathways (Nathan and Lal, 2020). Members of the 14-3-3 protein family also regulate innate immunity and participate in antiviral activity regulation through several signaling pathways (such as the MAPK, PI3K-Akt, NF-κB, and mTOR pathways) (Li et al., 2019a,b; Enchéry et al., 2021; Fu et al., 2021).

YWHAG belongs to the 14-3-3 protein family and is highly conserved across species (Cho and Park, 2020). Currently, YWHAG has been the most studied for its association with neurodegenerative diseases, cancer, and neurodevelopmental and neuropsychiatric disorders. Recent work has suggested that overexpression of the YWHAG protein in uterine leiomyoma cells has been reported to cause growth retardation and induce apoptosis (Shen et al., 2018), and miR-217 promotes the spread and invasion of glioblastoma by inhibiting YWHAG (Wang et al., 2017). However, the mechanism of YWHAG involvement in influenza virus infection remains unclear.

To address this gap in this study, we examined whether YWHAG interacts with viral M2 protein and affects IAV proliferation. Further, we examined the role of YWHAG in various steps of the viral life cycle and whether mutant YWHAG could affect viral M2.

## Materials and methods

### Plasmids, cells, and virus

Plasmids: Human YWHAG and open reading frame (ORF) of M2 derived from A/Puerto Rico/8/34 H1N1 (PR8) virus were cloned into p3Xflag-, p3Xmyc-, and PCAGGS-HA-expressing vector. Flag-tagged YWHAG(s) originated from synonymous mutation of Flag-tagged YWHAG. The mutant cDNAs, H158F and H195S, were generated by PCR with mutagenesis of the YWHAG sequences.

Cells: Madin-Darby canine kidney (MDCK) and A549 (human lung epithelial cell line) cells were purchased from the American Type Culture Collection (Manassas, VA, United States). Human embryonic kidney (HEK293T) and HeLa cells were maintained in our laboratory. A549 cells were cultured in Ham’s/F-12 (HyClone Laboratories, Logan, UT, United States) medium, whereas HEK293T cells and HeLa cells were grown in Dulbecco’s modified Eagle medium (DMEM)/high glucose (HyClone Laboratories) with 10% fetal bovine serum (FBS) and maintained at 37°C in 5% CO_2_.

Viruses: A/Puerto Rico/8/34 H1N1 (PR8) and A/duck/Hubei/WH18/2015 H5N6 (JX) are conserved in our laboratory. All experiments involving highly pathogenic avian influenza viruses were performed in a biosafety level 3 (BSL3) facility in accordance with the institutional biosafety manual.

### Co-immunoprecipitation assay

HEK293T cells were co-transfected with HA-tagged M2 and Flag-tagged YWHAG, or Flag-tagged M2 and HA-tagged YWHAG to examine the interaction of proteins. After 24 h of treatment with the cell lysis buffer for western blotting (WB) and immunoprecipitation (IP) containing phenylmethanesulfonyl fluoride (PMSF) for 10 min on ice, the cell lysates were collected by centrifugation and then incubated with anti-HA beads (MedChemExpress, Princeton, NJ, United States) at 25°C for 2 h or at 4°C overnight. Using a magnetic frame, the beads were collected and washed 4–5 times with ice-cold phosphate-buffered saline (PBS) with 0.5% Tween-20 (PBST). Immunoprecipitated proteins were extracted from the beads and 5 × loading buffer was added, followed by boiling in water for 10 min; the resulting samples were then subjected to SDS-PAGE and western blotting analysis.

### Fifty percent tissue culture infectious dose assay

MDCK cells were cultured in 96-well plates to perform the 50% tissue culture infectious dose (TCID_50_) assays according to standard procedures. Briefly, serial 10-fold dilutions of the virus supernatant were used for infecting MDCK cells in DMEM without FBS for 1 h at 37°C. The inoculum was removed, and the MDCK cells were washed with DMEM without FBS and then overlaid with 1% FBS DMEM containing 2 μg/ml N-tosyl-L-phenylalanine chloromethyl ketone (TPCK)-treated trypsin (Sigma, 4352157-1KT). After 2–3 days of incubation, the supernatants were analyzed using a hemagglutination inhibition test.

### Influenza A virus binding and entry assay

YWHAG-KO and control cells were seeded into 12-well plates at 5 × 10^5^ cells per well and cultured for 12 h. After 12 h, cells were infected with PR8 at a multiplicity of infection (MOI) = 5 and incubated at 4°C for 1 h. For the virus binding assay, cells were washed twice with cold PBS to remove unbound virus, cell lysates were harvested, and the amount of viral RNA (vRNA) and protein was determined using reverse transcription quantitative PCR (RT-qPCR) and western blot, respectively. For the virus entry assay, after incubating at 4°C for 1 h to allow for viral binding, infected cells were washed twice with cold PBS (4°C) to remove the unbound virus, and then incubated in prewarmed DMEM for 30 min at 37°C. Subsequently, cells were treated with neuraminidase for another 30 min at 37°C and washed three times with cold PBS (4°C) to remove non-internalized virions. Total viral RNA was extracted and quantified using RT-qPCR.

### Budding assay

YWHAG-KO or HEK293T cells were seeded in 100-mm cell culture dishes and infected with PR8 at MOI = 5 in YWHAG-KO cells or co-transfected with plasmids that express IAV M2 and YWHAG proteins in HEK293T cells. At 9 h after infection or 36 h after transfection, the culture supernatants were passed through a 0.45 mm filter, and virus particles were pelleted by ultracentrifugation through a 20% sucrose cushion (110,000 × *g* for 3 h at 4°C, Beckman SW32Ti rotor, Beckman Coulter, Pasadena, CA, United States). After centrifugation, 5 × loading buffer was added to the solution. Western blotting was used to determine the M2 protein content.

### Electron microscopy

For thin-section electron microscopy, PR8 IAV-infected HEK293T cells containing Flag-tagged YWHAG or YWHAG KO cells were scraped off the plates. The cells were then centrifuged at 4,000 × *g* for 10 min. Cells were then fixed with 2.5% glutaraldehyde at 4°C for at least 4 h and then stained with 1% osmium tetroxide for 30 min at room temperature. Subsequently, the specimens were dehydrated with increasing concentrations of ethanol. The cells were embedded in epoxy resin; the resin blocks were sliced into 80-nm-thick sections, placed on copper grids, and stained with 2% aqueous uranyl acetate and Reynold’s lead citrate. Transmission electron microscopy was used to observe the negatively stained ultrathin sections.

### Immunofluorescence and confocal microscopy

HeLa cells were transfected with HA-tagged M2 along with Flag-tagged YWHAG or empty vector for 24 h, and the cells were fixed with 4% paraformaldehyde, permeabilized with 0.1% Triton X-100, and sealed with 2% BSA. Cells were then incubated with rabbit anti-HA (1:200 dilution) and mouse anti-Flag (1:200 dilution) antibodies, followed by incubation with anti-rabbit Alexa Fluor 594-conjugated antibody (1:500 dilution) and anti-mouse Alexa Fluor 488-conjugated antibody (1:500 dilution). Subsequently, the cells were incubated with DAPI (1:1,000) for 10–15 min to stain the nuclei. Images were recorded using a Zeiss LSM 880 confocal fluorescence microscope (Carl Zeiss AG, Jena, Germany).

### Statistics

Data plotting and analyses were performed using GraphPad Prism software with Student’s *t*-tests (GraphPad Software, San Diego, CA, United States). Data are shown as the mean ± standard deviation (SD) derived from three independent experiments. Statistical significance was set at *p* < 0.05.

## Results

### YWHAG interacts with influenza A virus matrix protein 2

**FIGURE 1:**
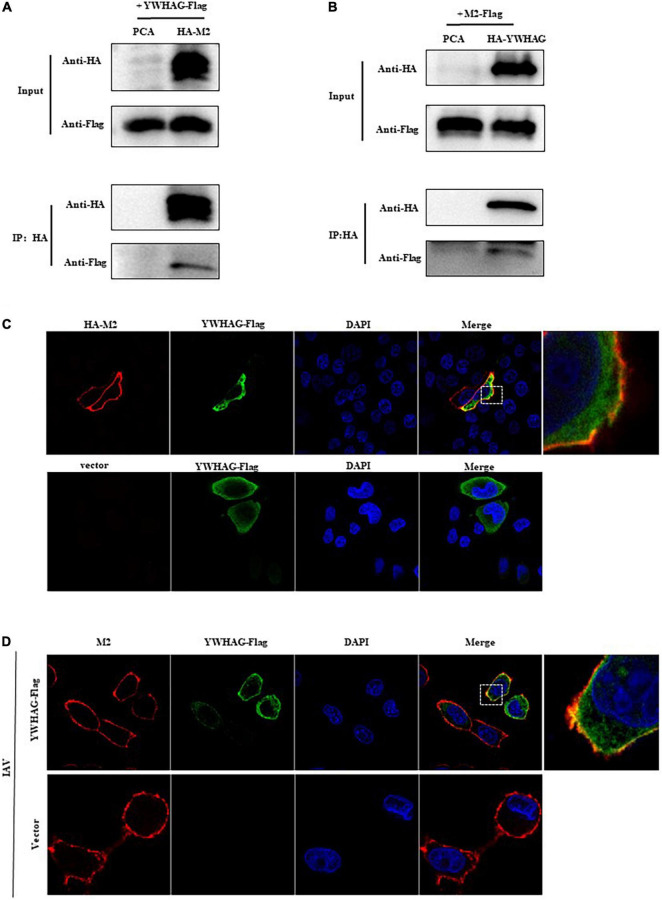
The host factor YWHAG interacts with M2. **(A,B)** Flag-tagged YWHAG-transfected with empty vector or HA-tagged M2 in HEK293T cells. **(A)** Flag-tagged M2 co-transfected with empty vector or HA-tagged YWHAG in HEK293T cells. **(B)** Cell lysates were incubated with anti-HA beads for 2 h at 25°C or at 4°C overnight. The beads were washed with PBST 4–5 times and IP eluates were subjected to western blotting analysis. **(C)** HeLa cells were co-transfected HA-tagged M2 and Flag-tagged YWHAG or empty vector, the cells were fixed and stained with mouse anti-Flag mAb and rabbit anti-HA pAb, and then incubated with Alexa Fluor 488 anti-mouse IgG (H + L) (green) and Alexa Fluor 594 goat anti-Rabbit IgG (H + L) (red). The nuclei were stained with DAPI. **(D)** Flag-tagged YWHAG or empty vector was transfected into HeLa cells and infected with PR8 at MOI = 1. The anti-M2 rabbit red fluorescent antibody and anti-Flag mouse green fluorescent antibody were used for staining. The interaction part enclosed by the white box was enlarged, as shown in the right.

The host protein YWHAG was identified by immunoprecipitation united with mass spectrometry (IP–MS) (Supplementary Figure 1 and Supplementary Table 1). To verify the interaction between M2 and YWHAG, HA-tagged M2 and Flag-tagged YWHAG were co-transfected into HEK293T cells, followed by a co-immunoprecipitation assay using anti-HA polyclonal antibody (pAb) or anti-FLAG monoclonal antibody (mAb) in which empty vectors were used as controls. The results showed that Flag-tagged YWHAG could be precipitated with an anti-HA pAb upon co-transfection with HA-tagged M2, but not with the empty vector (Figure 1A). When a reverse Co-IP experiment was employed with an anti-HA pAb, Flag-tagged M2 was also co-immunoprecipitated with HA-tagged YWHAG (Figure 1B) and during IAV infection, endogenous YWHAG could be precipitated with an anti-M2 pAb (Supplementary Figure 2A), further demonstrating the specificity of the interaction between the host factor YWHAG and the viral M2 protein. The localization of M2 and YWHAG was examined by immunofluorescence and confocal microscopy in HeLa cells transfected with Flag-tagged YWHAG and HA-tagged M2 or empty vector (Figure 1C). The co-localization of YWHAG with M2 was further confirmed in PR8 virus-infected HeLa cells containing Flag-tagged YWHAG or empty vector (Figure 1D) and in A549 cells (Supplementary Figure 2B). Notably, M2 and YWHAG were clearly co-localized in the cell membrane region upon co-expression. These results demonstrate that YWHAG interacts with viral M2, and suggest a potential function of YWHAG in influenza virus infection.

### YWHAG inhibits influenza A virus replication

**FIGURE 2:**
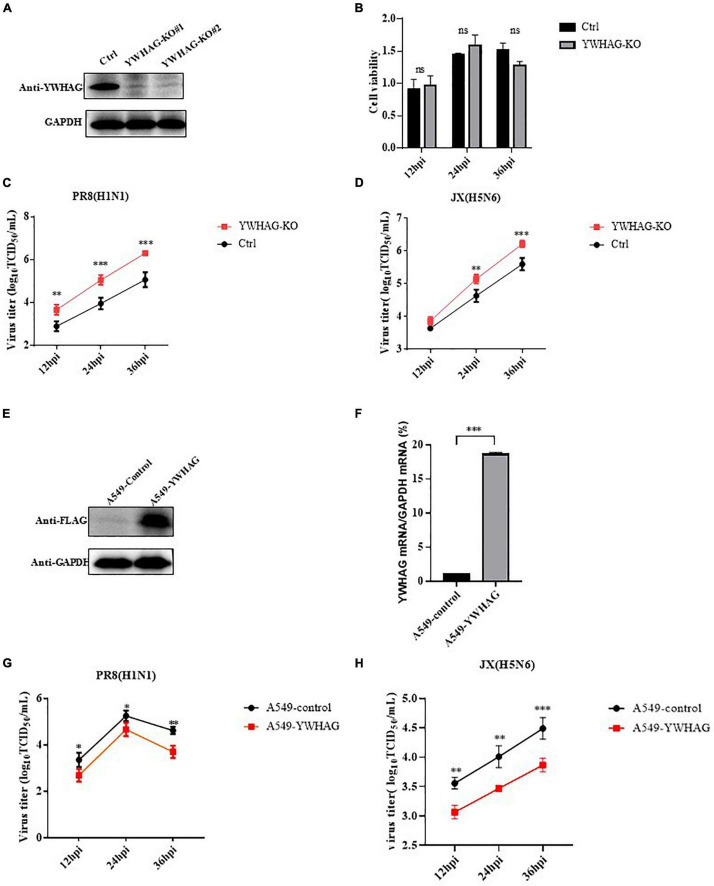
YWHAG inhibits influenza A virus (IAV) replication. **(A)** Endogenous YWHAG in A549 cells was knocked out using the CRISPR/Cas9 system. YWHAG expression was determined by western blotting. **(B)** Viability of YWHAG-KO cells was measured using a CCK8 assay. The data are presented as mean ± standard deviation (*SD*). **(C,D)** Control (Ctrl) or YWHAG-knockout (YWHAG-KO) A549 cells were infected with PR8 (H1N1) **(C)** or JX (H5N6). **(D)** The supernatants were harvested and viral titers were determined at the indicated times. Stable overexpression of Flag-tagged YWHAG was confirmed by western blotting using a mouse anti-Flag mAb **(E)** and reverse-transcription quantitative PCR (RT-qPCR) **(F)** compared with that in control cells transduced with an empty retrovirus. **(G,H)** YWHAG-overexpressing cells or control cells were infected with PR8 (H1N1) **(G)**, JX (H5N6) **(H)** at MOI = 0.01. Supernatants were collected at different times, and virus titers were determined using a TCID_50_ assay on MDCK cells. The data are presented as the means ± SD by *t*-test (*p < 0.05; **p < 0.01; ***p < 0.001).

To study the role of YWHAG during the viral life cycle, we analyzed the effect of YWHAG on viral replication. We first examined IAV whether affected the expression of YWHAG and found that there was no change by western blotting and RT-qPCR (Supplementary Figure 3). Then, we generated YWHAG-KO A549 cell line by using the CRISPR/Cas9 system. Knockout of YWHAG was confirmed by western blot using rabbit anti-YWHAG pAb (Figure 2A) and YWHAG-KO had no obvious effect on cell viability, as measured using the CCK8 assay (Figure 2B). Next, we infected YWHAG-KO or control cells with PR8 virus at MOI = 0.01, the supernatants were collected at 12, 24, and 36 h post-infection (p.i.) and titrated on MDCK cells. The titers of PR8 virus in YWHAG-KO cells were dramatically increased compared with those in the control cells (Figure 2C). Similar results were observed for influenza virus JX (Figure 2D). We further constructed a stable YWHAG-overexpressing cell line with a retrovirus encoding Flag-tagged YWHAG or that with an empty vector as a control cell line. As expected, expression of YWHAG at both mRNA and protein levels was higher in YWHAG-overexpressing cells than in control cells (Figures 2E,F). Control and YWHAG-overexpressing A549 cells were infected with PR8 and JX at MOI = 0.01. Culture supernatants were then collected at different time points after infection and titrated against MDCK cells. Strikingly, the viral titers from YWHAG-overexpressing cells showed a significant decrease at 12–36 h p.i. (Figures 2G,H). Interestingly, we reconstituted plasmid of expressed Flag-tagged YWHAG(s) by the synonymous mutation and transfected Flag-tagged YWHAG(s) and empty vector into YWHAG-KO cells or control cells, following infection with PR8 MOI = 0.01 at 12–36 h p.i. The result of TCID_50_ assays demonstrated significantly reduced after transfection of Flag-tagged YWHAG(T) into the YWHAG-KO cells compared with empty vector into the YWHAG-KO cells (Supplementary Figure 4). These findings indicate that YWHAG negatively modulates IAV replication.

### YWHAG does not affect the binding, entry, replication, and transcription in early influenza A virus infection

**FIGURE 3:**
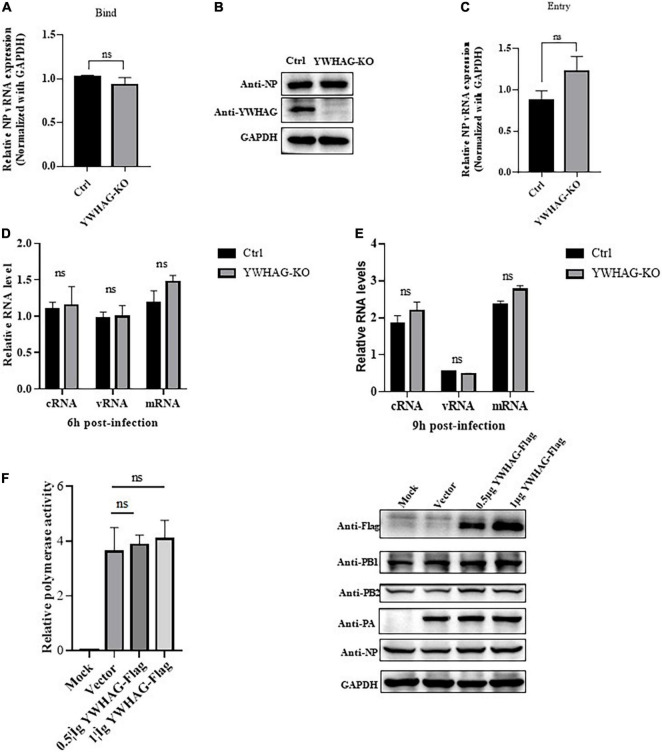
YWHAG does not affect the binding, entry, replication, and transcription of IAV in the early stages of infection. **(A,B)** YWHAG-KO or control cells were incubated with PR8 at MOI = 5, at 4°C for 1 h, the viral copy number of bound viruses in the cell lysates was assessed using reverse-transcription quantitative PCR (RT-qPCR) **(A)** and western blotting **(B)**. **(C)** YWHAG-KO and control cells were incubated with PR8 at an MOI = 5 at 4°C for 1 h and then allowed to internalize bound IAV by incubation at 37°C for 30 min, followed by the addition of exogenous NA to remove the cell-surface virions and incubation for another 30 min. Viral entry was measured using reverse-transcription quantitative PCR (RT-qPCR). **(D,E)** YWHAG-KO or control cells were infected with PR8 at MOI = 5. Total RNA was harvested at 6 h **(D)** and 9 h **(E)** post-infection, and the nucleoprotein (NP) in vRNA, cRNA, and mRNA was analyzed using reverse-transcription quantitative PCR (RT-qPCR). **(F)** HEK293T cells were co-transfected with pPolI-Luc and expression plasmids containing viral PB1, PB2, PA, or NP of H1N1 together with an empty vector or Flag-tagged YWHAG. At 24 h post-transfection, western blotting and a dual-luciferase assay were performed wherein the relative firefly luciferase activity was normalized to that of the internal control.

To explore how YWHAG negatively regulates the replication of IAV, we first examined whether YWHAG affected viral binding or entry. YWHAG-KO and control A549 cells were incubated with PR8 at MOI = 5 for 1 h at 4°C to allow virus binding, followed by incubation with prewarmed DMEM at 37°C to allow virus entry. RT-qPCR (Figure 3A) and western blot (Figure 3B) or entry assay (Figure 3C) showed that YWHAG did not affect virus binding and entry. We then investigated whether YWHAG affected viral transcription and replication. YWHAG-KO and control cells were infected with PR8 at MOI = 5. At 6 and 9 h p.i., vRNA, mRNA, and cRNA derived from the nucleoprotein (NP) were measured using RT-qPCR (Figures 3D,E). At both time points, the levels of all three species of RNA were not conspicuous in the YWHAG-KO cells compared with those in the control cells. We further determined whether YWHAG regulates viral RNA synthesis by examining polymerase activity. HEK293T cells were co-transfected with Flag-tagged YWHAG and polymerase plasmids, including PB1, PB2, PA, and NP, as well as a pPolI-driven RNA expression plasmid. The data showed that YWHAG overexpression resulted in no obvious difference in polymerase activity (Figure 3F). Overall, these data suggest that YWHAG has no effect on the early stages of viral infection, including viral binding, entry, viral RNA transcription, and replication.

### YWHAG suppresses matrix protein 2-mediated budding of influenza A virus

**FIGURE 4:**
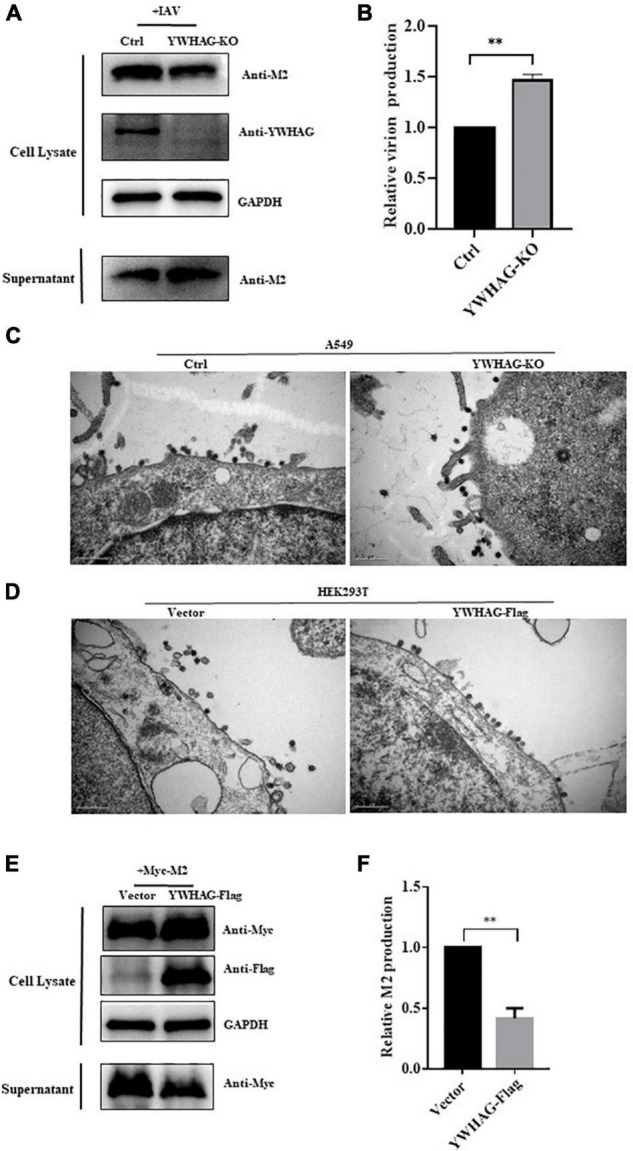
YWHAG suppresses M2-mediated budding of IAV. **(A,B)** YWHAG-KO or control cells were infected with PR8 at MOI = 5 for 10 h and the supernatants were collected by ultracentrifugation. Cell lysates and virions were determined by western blotting **(A)**. Relative virions production was calculated based on the M2 protein band intensities and expressed as the ratios of viral lysate/cell lysate **(B)**. The level of virions from control cells was set as 100%. **(C,D)** YWHAG-KO or control cells **(C)** and HEK293T cells transfected with Flag-tagged YWHGA or empty vector **(D)** were infected with PR8 at MOI = 5 for 10 h; thin sections were prepared and analyzed by electron microscopy. Scale bars indicate 500 nm. **(E,F)** HEK293T cells were transfected with Myc-tagged M2 and Flag-tagged YWHAG. The culture supernatants were harvested by ultracentrifugation. Cell lysates and centrifuged supernatants samples were examined by western blotting **(E)**. The intensities of relative M2 production were determined using Image J **(F)**. The data are presented as the means ± SD by *t*-test (**p < 0.01).

During IAV infection, M2 protein is involved in viral membrane scission and allows the formation and release of progeny IAV virions. We thus explored whether YWHAG affects IAV budding. YWHAG-KO or control cells were infected with PR8 at MOI = 5 for 10 h and the virions in the supernatants were collected by ultracentrifugation. The results showed a 1.5-fold increase in particle-associated M2 in YWHAG-KO cells compared with control cells (Figures 4A,B). YWHAG-KO cells or Flag-tagged YWHAG-transfected HEK293T cells were infected with PR8 at MOI = 5. After 10 h, we examined the budding of influenza viruses by transmission electron microscopy. In YWHAG-KO cells, budding of IAV virions was observed far from the cell surface compared with that of the control cells (Figure 4C). HEK293T cells transfected with Flag-tagged YWHAG presented IAV virions in the process of budding but blocked particle release (Figure 4D). Considering that YWHAG interacted with M2 protein and M2 had an important role in the budding of IAV. We determined whether YWHAG participates in the budding of M2. HEK293T cells were transfected with Myc-tagged M2 and Flag-tagged YWHAG or empty vector, supernatants and cells were then collected 36 h after treatment. Western blotting and statistical analysis of relative M2 production showed that co-transfection of M2 and YWHAG impaired M2 release (Figures 4E,F). Taken together, these results indicate that YWHAG suppresses M2-mediated budding of IAV.

### YWHAG with H158F mutation inhibits influenza A virus matrix protein 2 budding

**FIGURE 5:**
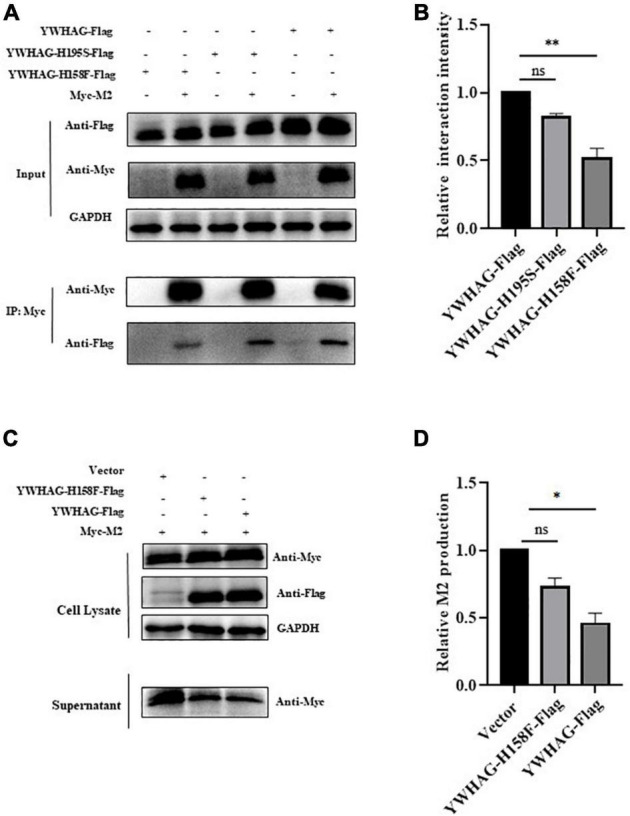
YWHAG with H158F mutation inhibits M2 budding. **(A)** HEK293T cells were transfected to produce Flag-tagged YWHAG protein variants together with the indicated Myc-tagged M2. Protein interactions were detected using co-immunoprecipitation. **(B)** Relative interaction intensity was calculated based on protein band intensities and expressed as the ratios of IP (Flag/Myc). **(C,D)** HEK293T cells were transfected with Myc-tagged M2 and Flag-tagged YWHAG variants. Culture supernatants were harvested by ultracentrifugation. Cell lysates and supernatant samples were examined using western blotting **(C)** and the relative M2 production was analyzed **(D)**. The data are presented as the means ± SD by *t*-test (*p < 0.5; **p < 0.01).

Given that YWHAG was the peripheral protein, it could affect the binding ability of the membrane through electrostatic distribution, and the H158 and H195 residues of YWHAG play important roles in its membrane affinity. We explored whether H158F and H195S mutations affect the interaction of YWHAG with M2. Flag-tagged YWHAG or its mutants were co-transfected with Myc-tagged M2 in HEK293T cells. The results showed that the interaction between YWHAG and M2 was substantially reduced with the H158F mutant compared with the H195S mutant and wild-type Flag-tagged YWHAG (Figures 5A,B). Similarly, we explored whether the H158F mutation affected the budding of M2. HEK293T cells were thus transfected with Myc-tagged M2 and Flag-tagged H158F-YWHAG, YWHAG, or empty vector; the culture supernatants and cells were then collected after 36 h. Relative M2 production showed a reduction of M2 was blocked compared with YWHAG by western blot (Figures 5C,D). These data indicate that the H158F mutation inhibited the interaction between YWHAG and M2 and its budding.

## Discussion

IAV forms virions through a series of processes, including binding, entry, and vRNP assembly, and releasing on the cell membrane (Luo, 2012; Pleschka, 2013). M2 protein shows an important function in the influenza virus life cycle. On the one hand, M2 as an ion channel protein is activated during influenza virus internalization. The internal environment of the virus is acidic, which is conducive to the separation of M1 and vRNPs (Nayak et al., 2009). On the other hand, when the virus particles are released, influenza virus does not need to go through the host endosomal sorting complex required for transport (ESCRT) machinery like other capsular viruses but involves the interaction of M1, HA, NA, and M2 (Rossman et al., 2010; Hutchinson, 2018). M1 is a bridge that connects M2, HA, and NA (Peukes et al., 2020). M2 protein is recruited to the cell membrane to mediate membrane scission, and sialic acid connected with HA is destroyed by NA to release the virion (Moreira et al., 2021). In these processes, the viral proteins as well as a large number of host factors are required to mediate the ion channel activity of M2 and its membrane scission. For example, IAV-induced autophagy is dependent on M2 ion channel activity, and SERCA is proportional to autophagic flux, therefore, IAV M2 induces autophagic flux arrest by modulating host factor SECRA activity (Peng et al., 2021). The host factor AnX6 disrupts virus release by interacting with the M2 CT region (Musiol et al., 2013). The host factor BST-2 interacts with M2 protein to reduce the formation of VLPs and causes BST-2 protein degradation by proteases (Hu et al., 2017). MAHRC8 targets M2 proteins and inhibits M2 release from the cell membrane (Martyna et al., 2020; Liu et al., 2021b). In this study, we verified the interaction between the host factor YWHAG and influenza virus M2 protein by co-immunoprecipitation and found that YWHAG interacts with M2 mostly on the cell membrane as determined using confocal microscopy. Further exploration of the effect of YWHAG on the replication of IAV revealed that YWHAG does not affect the proliferation of IAV through virus binding, invasion, and RNP assembly but affects the emergence of influenza virus. Interestingly, YWHAG inhibits influenza virus production by affecting the release of M2. This also coincides with the co-location of YWHAG and M2 on the cytoplasmic membrane (the position where the virus buds).

The 14-3-3 of family proteins are ubiquitous regulatory proteins in eukaryotes that play important roles in several biological processes (Fu et al., 2000), including the cell cycle, development, DNA damage, apoptosis, cytoskeletal rearrangement, protein transport, and signal transduction, among others (Sluchanko and Gusev, 2010; Obsil and Obsilova, 2011; Cau et al., 2018). These functions are mainly related to their phosphorylation via ligand binding (Fu et al., 2000; de Boer et al., 2013). In viral infections, the 14-3-3 proteins also play an important role in binding to viral proteins through phosphorylation (Liu et al., 2021a). Take an example, after a phosphorylation mutation at site 369 of the parainfluenza virus M protein, it no longer binds to YWHAB to promote VLP production (Pei et al., 2011). And the NS3 protein of Zika virus increases viral production by competitively binding the phosphorylation mode motifs of YWHAE and YWHAH, which blocks the transfer of RIG and MAVS, respectively (Riedl et al., 2019). However, in our study, we found that YWHAG affected the budding of influenza virus M2 was not by impacting the phosphorylation of IAV M2 (Supplementary Figure 5). Because YWHAG, a peripheral protein, can affect the binding ability of the membrane through electrostatic distribution (Lee et al., 2005) and interact with M2 in the cell membrane that was location of budding of M2. We speculated that YWAHG may affect the release of M2 by influencing the affinity of M2 with the membrane, as well as Bustad et al. (2012) reported that His158 and His195 of YWHAG, especially His195, are crucial for ligand-induced membrane interactions. Therefore, we chose H158 and H195 of YWHAG to explore YWHAG how to affect the budding of M2. As expected, we introduced mutations at these two sites and found that they indeed affected the interaction with viral M2 and the budding of M2. However, mutation of the His158 site had a greater effect. The reasons for this phenomenon are still unclear and require further exploration.

## Conclusion

In conclusion, our study revealed that YWHAG interacts with influenza virus M2 and influences influenza virus replication by affecting the budding of M2. Thus, it enhances our understanding of the relationship between influenza virus and host proteins.

## Data Availability Statement

The original contributions presented in this study are included in the article/Supplementary Material, further inquiries can be directed to the corresponding author/s.

## Author contributions

HM, KH, and MJ conceived and designed the experiments. HM and LC performed the experiments. HM, KH, and XL analyzed the data. TX contributed to the reagents and materials. HM and KH prepared the manuscript. All authors contributed to the manuscript and approved the submitted version.

## Conflict of Interest

The authors declare that the research was conducted in the absence of any commercial or financial relationships that could be construed as a potential conflict of interest.

## Publisher’s Note

All claims expressed in this article are solely those of the authors and do not necessarily represent those of their affiliated organizations, or those of the publisher, the editors and the reviewers. Any product that may be evaluated in this article, or claim that may be made by its manufacturer, is not guaranteed or endorsed by the publisher.
