# Supplementary figures and images for: YWHAG inhibits influenza a virus replication by suppressing the release of viral M2 protein
Source: Front Microbiol. 2022 Jul 19;13:951009. doi: 10.3389/fmicb.2022.951009 (PMC9343881; doi:10.3389/fmicb.2022.951009)

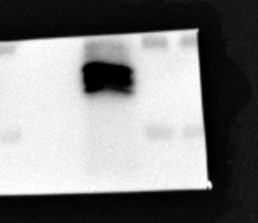

Supplement: Supplementary file 1 [file Data_Sheet_1.ZIP › Raw date/Figure1/Figure1A/IP-HA-M2.jpg]

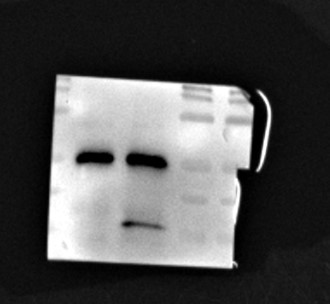

Supplement: Supplementary file 1 [file Data_Sheet_1.ZIP › Raw date/Figure1/Figure1A/IP-YWHAG-Flag.jpg]

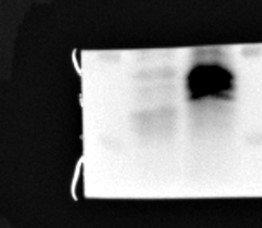

Supplement: Supplementary file 1 [file Data_Sheet_1.ZIP › Raw date/Figure1/Figure1A/Input-HA-M2.jpg]

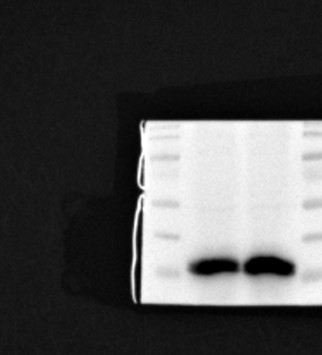

Supplement: Supplementary file 1 [file Data_Sheet_1.ZIP › Raw date/Figure1/Figure1A/Input-YWHAG-Flag.jpg]

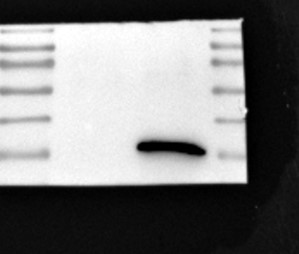

Supplement: Supplementary file 1 [file Data_Sheet_1.ZIP › Raw date/Figure1/Figure1B/IP-HA-YWHAG.jpg]

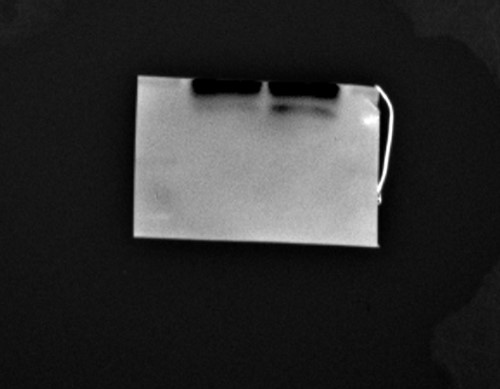

Supplement: Supplementary file 1 [file Data_Sheet_1.ZIP › Raw date/Figure1/Figure1B/IP-M2-Flag.jpg]

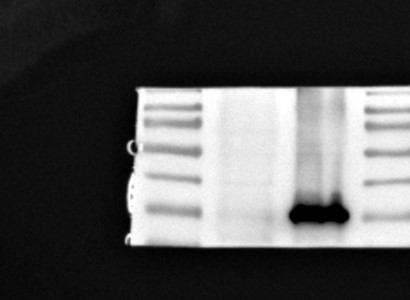

Supplement: Supplementary file 1 [file Data_Sheet_1.ZIP › Raw date/Figure1/Figure1B/Input-HA-YWHAG.jpg]

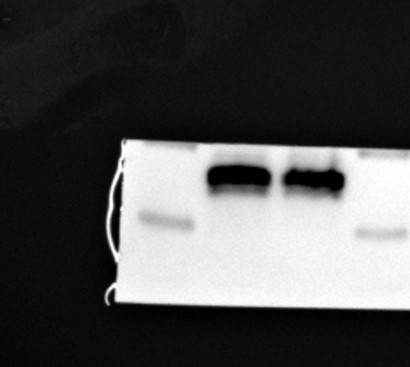

Supplement: Supplementary file 1 [file Data_Sheet_1.ZIP › Raw date/Figure1/Figure1B/Input-M2-Flag.jpg]

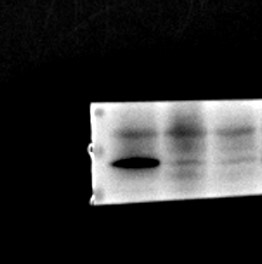

Supplement: Supplementary file 1 [file Data_Sheet_1.ZIP › Raw date/Figure2/Figure2A/Anti-YWHAG.jpg]

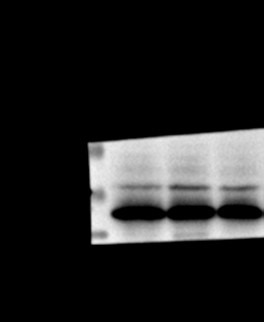

Supplement: Supplementary file 1 [file Data_Sheet_1.ZIP › Raw date/Figure2/Figure2A/GAPDH.jpg]

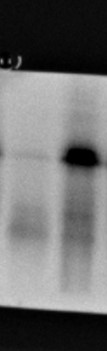

Supplement: Supplementary file 1 [file Data_Sheet_1.ZIP › Raw date/Figure2/Figure2E/Anti-Flag.jpg]

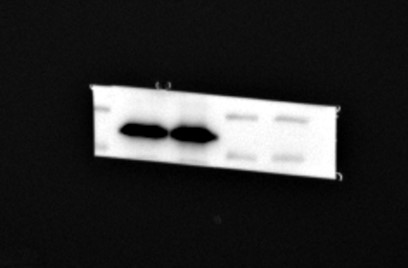

Supplement: Supplementary file 1 [file Data_Sheet_1.ZIP › Raw date/Figure2/Figure2E/GAPDH.jpg]

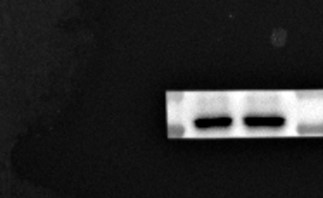

Supplement: Supplementary file 1 [file Data_Sheet_1.ZIP › Raw date/Figure3/Figure3B/Anti-NP.jpg]

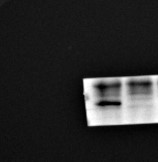

Supplement: Supplementary file 1 [file Data_Sheet_1.ZIP › Raw date/Figure3/Figure3B/Anti-YWHAG.jpg]

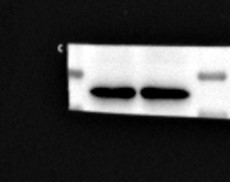

Supplement: Supplementary file 1 [file Data_Sheet_1.ZIP › Raw date/Figure3/Figure3B/GAPDH.jpg]

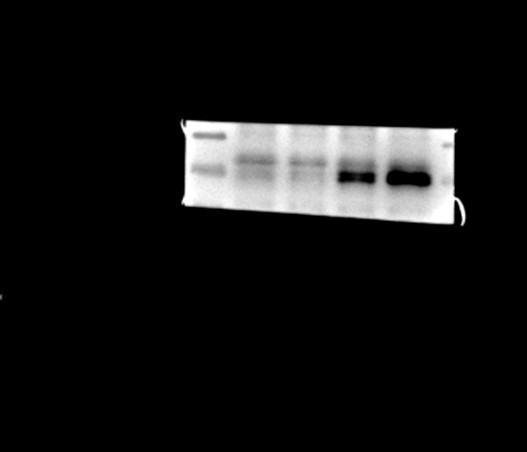

Supplement: Supplementary file 1 [file Data_Sheet_1.ZIP › Raw date/Figure3/Figure3F/Anti-Flag.jpg]

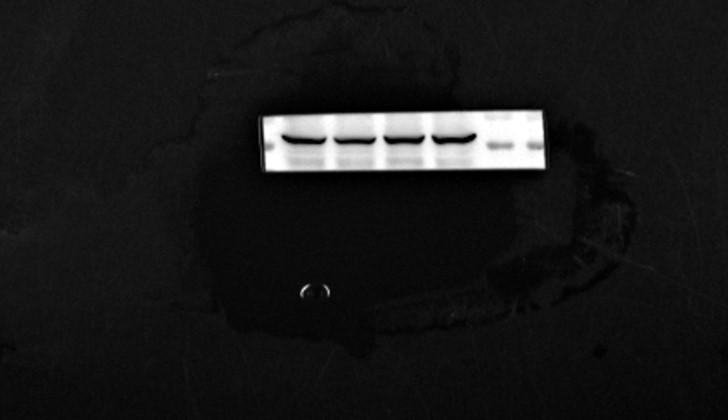

Supplement: Supplementary file 1 [file Data_Sheet_1.ZIP › Raw date/Figure3/Figure3F/Anti-NP.jpg]

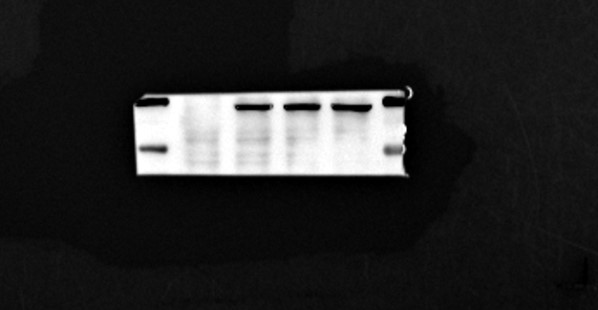

Supplement: Supplementary file 1 [file Data_Sheet_1.ZIP › Raw date/Figure3/Figure3F/Anti-PA.jpg]

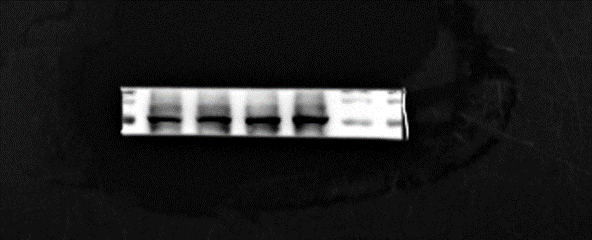

Supplement: Supplementary file 1 [file Data_Sheet_1.ZIP › Raw date/Figure3/Figure3F/Anti-PB1.jpg]

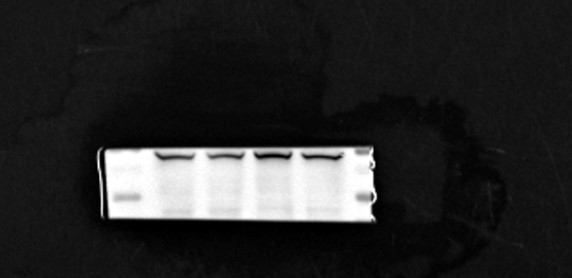

Supplement: Supplementary file 1 [file Data_Sheet_1.ZIP › Raw date/Figure3/Figure3F/Anti-PB2.jpg]

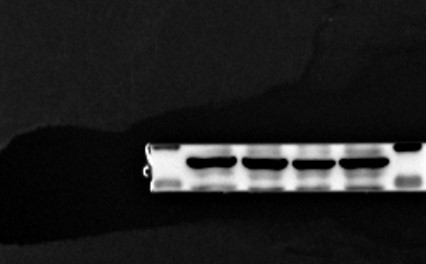

Supplement: Supplementary file 1 [file Data_Sheet_1.ZIP › Raw date/Figure3/Figure3F/GAPDH.jpg]

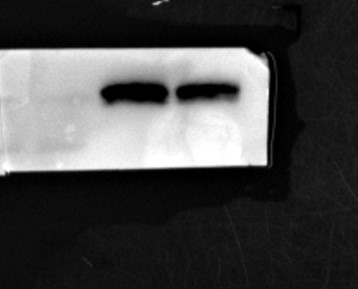

Supplement: Supplementary file 1 [file Data_Sheet_1.ZIP › Raw date/Figure4/Figure4A/Anti-M2-Cell Lysate.jpg]

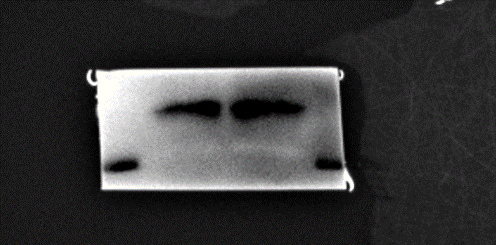

Supplement: Supplementary file 1 [file Data_Sheet_1.ZIP › Raw date/Figure4/Figure4A/Anti-M2-VLP.jpg]

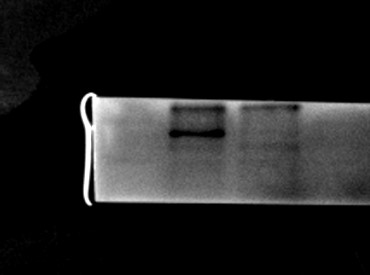

Supplement: Supplementary file 1 [file Data_Sheet_1.ZIP › Raw date/Figure4/Figure4A/Anti-YWHAG-Cell Lysate.jpg]

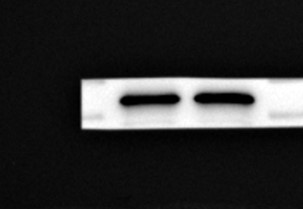

Supplement: Supplementary file 1 [file Data_Sheet_1.ZIP › Raw date/Figure4/Figure4A/GAPDH.jpg]

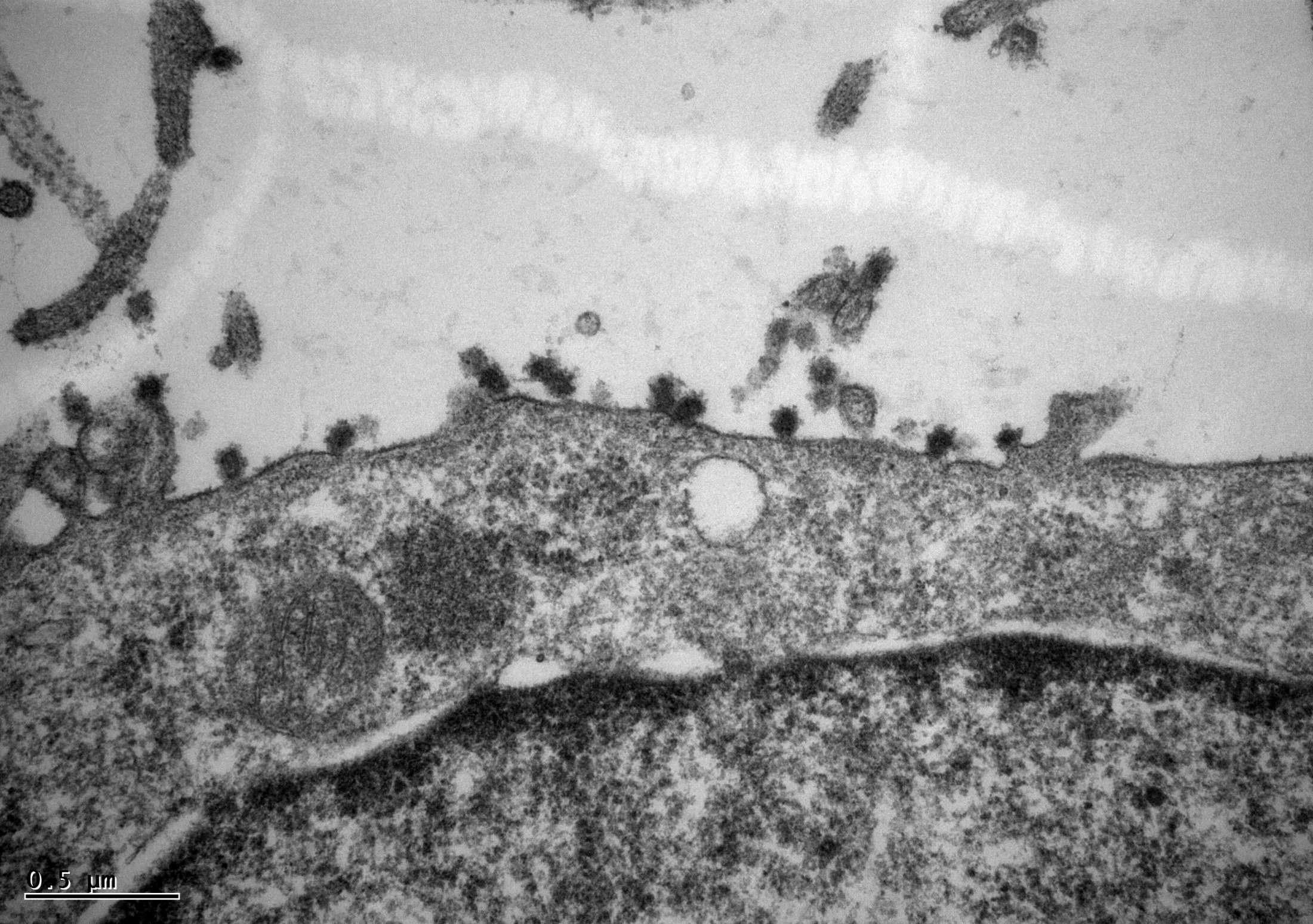

Supplement: Supplementary file 1 [file Data_Sheet_1.ZIP › Raw date/Figure4/Figure4C/Figure4C-Ctrl.jpg]

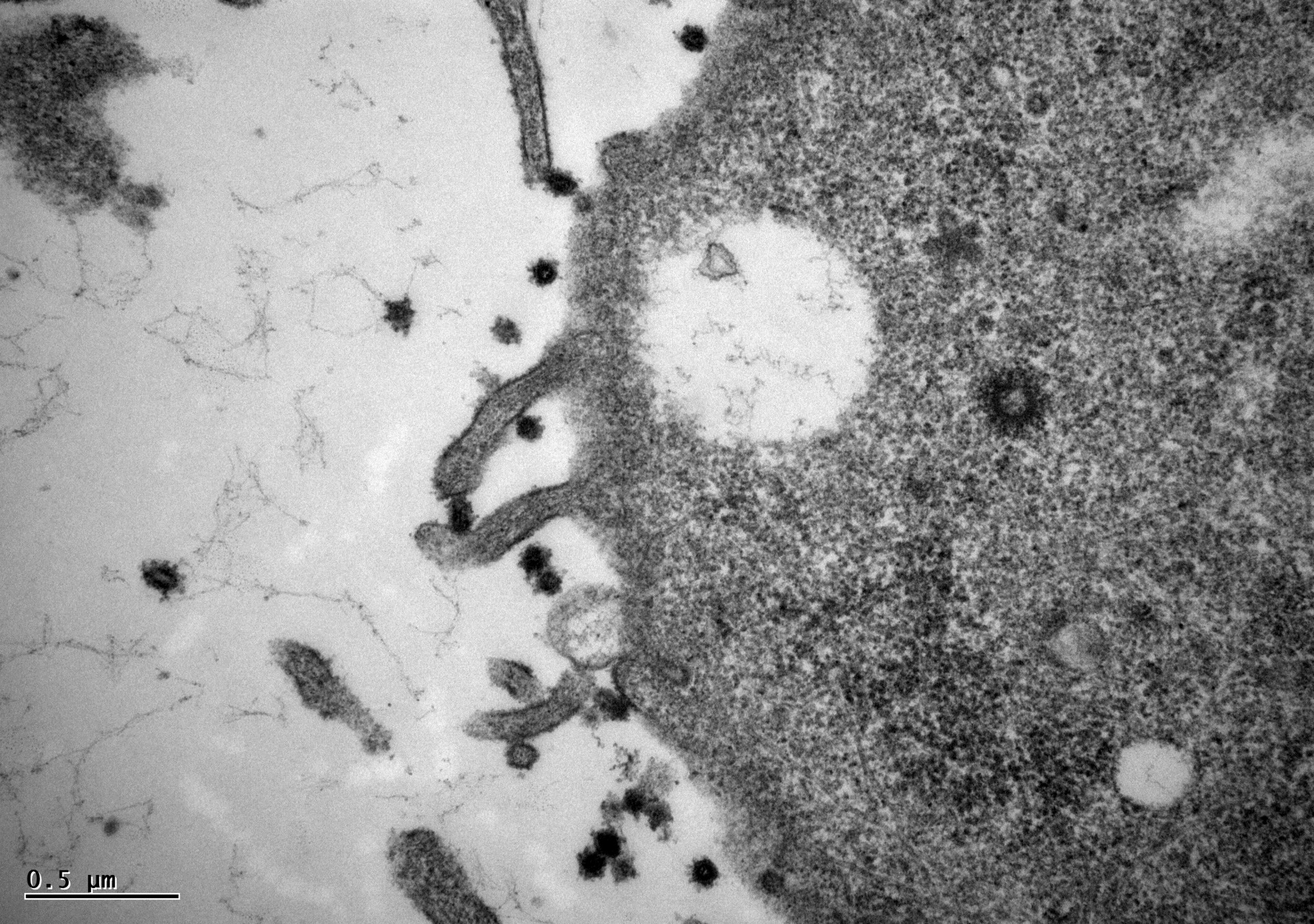

Supplement: Supplementary file 1 [file Data_Sheet_1.ZIP › Raw date/Figure4/Figure4C/Figure4C-YWHAG-KO.jpg]

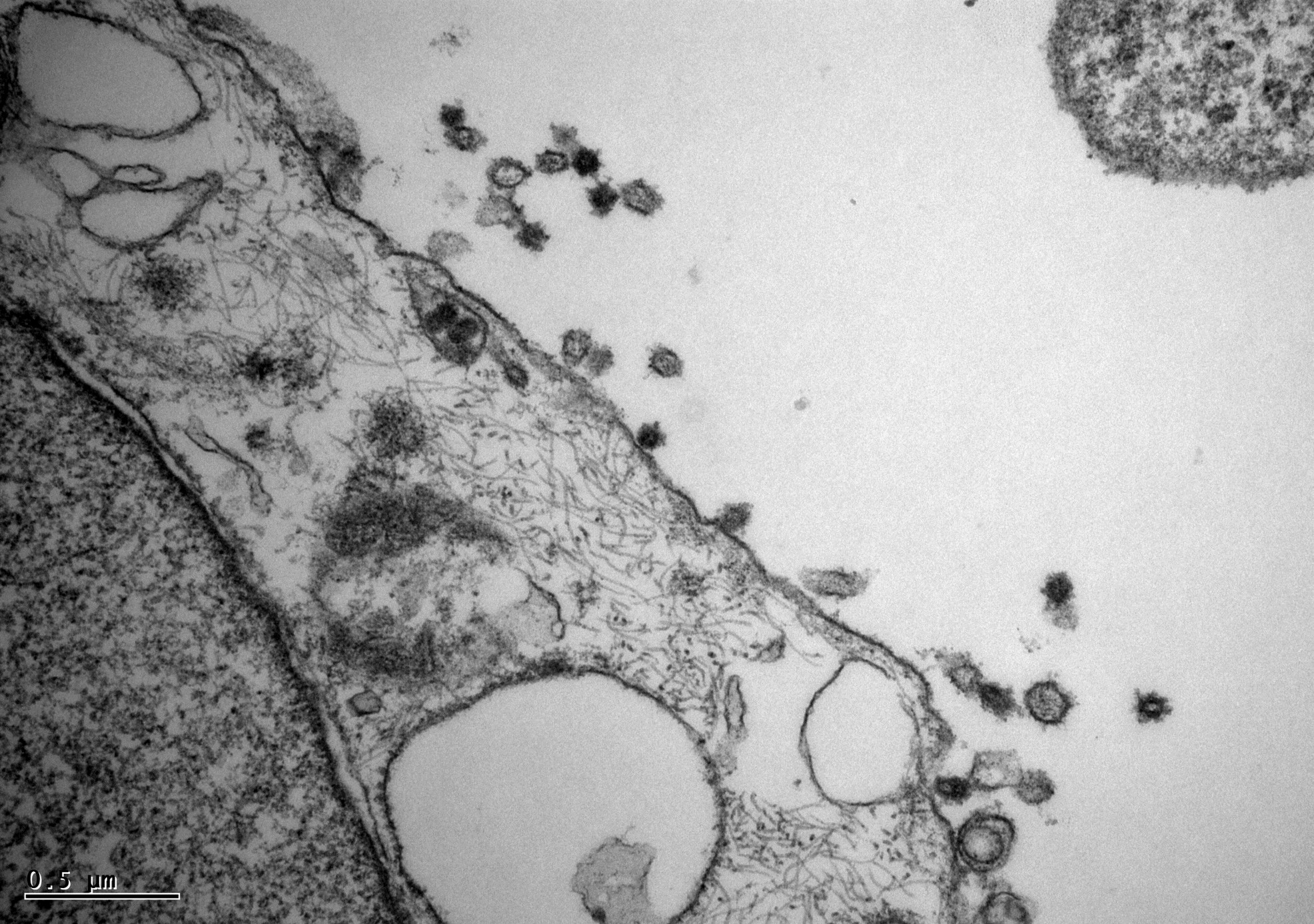

Supplement: Supplementary file 1 [file Data_Sheet_1.ZIP › Raw date/Figure4/Figure4D/Figure4D-Vector.jpg]

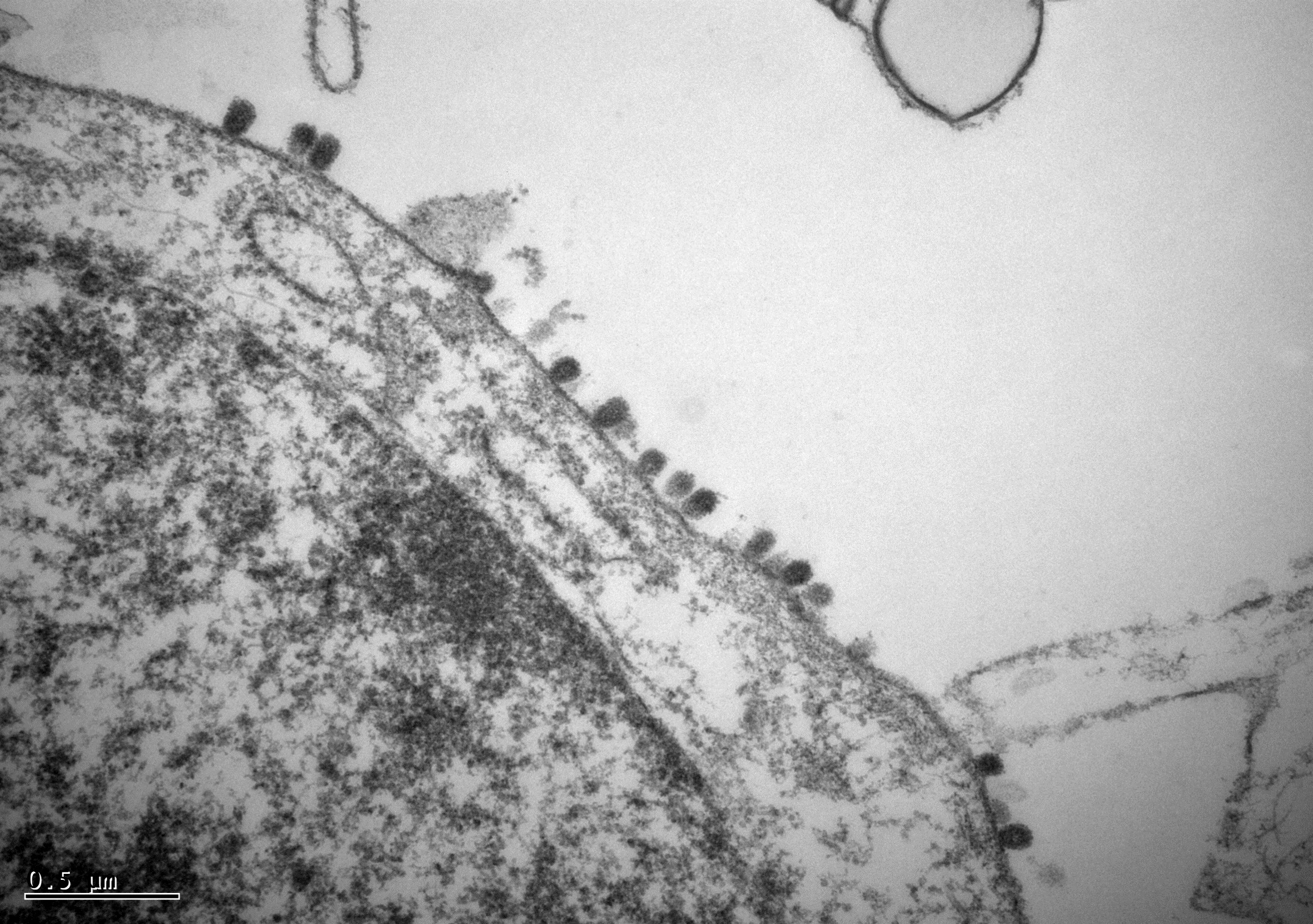

Supplement: Supplementary file 1 [file Data_Sheet_1.ZIP › Raw date/Figure4/Figure4D/Figure4D-YWHAG-Flag.jpg]

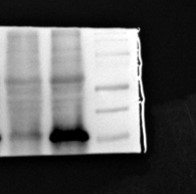

Supplement: Supplementary file 1 [file Data_Sheet_1.ZIP › Raw date/Figure4/Figure4E/Anti-Flag-Cell Lysate.jpg]

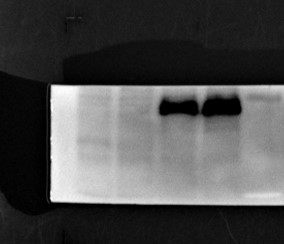

Supplement: Supplementary file 1 [file Data_Sheet_1.ZIP › Raw date/Figure4/Figure4E/Anti-Myc-Cell Lysate.jpg]

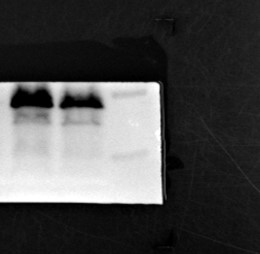

Supplement: Supplementary file 1 [file Data_Sheet_1.ZIP › Raw date/Figure4/Figure4E/Anti-Myc-VLP.jpg]

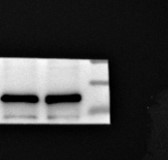

Supplement: Supplementary file 1 [file Data_Sheet_1.ZIP › Raw date/Figure4/Figure4E/GAPDH.jpg]

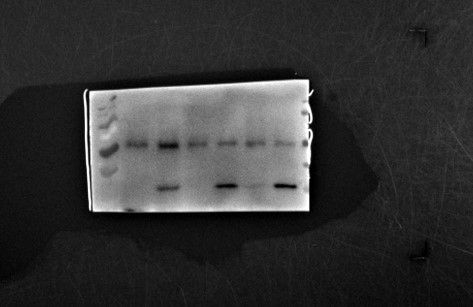

Supplement: Supplementary file 1 [file Data_Sheet_1.ZIP › Raw date/Figure5/Figure5A/Anti-Flag-IP.jpg]

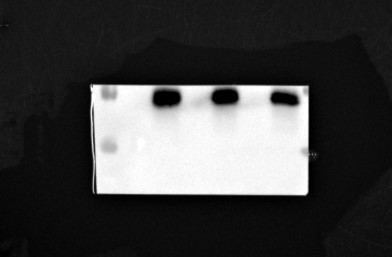

Supplement: Supplementary file 1 [file Data_Sheet_1.ZIP › Raw date/Figure5/Figure5A/Anti-Myc-IP.jpg]

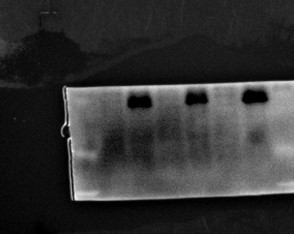

Supplement: Supplementary file 1 [file Data_Sheet_1.ZIP › Raw date/Figure5/Figure5A/Anti-Myc-input.jpg]

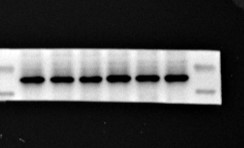

Supplement: Supplementary file 1 [file Data_Sheet_1.ZIP › Raw date/Figure5/Figure5A/GAPDH.jpg]

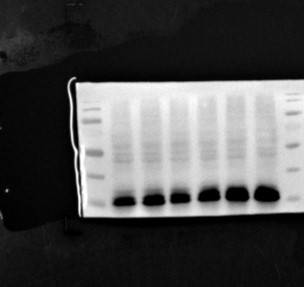

Supplement: Supplementary file 1 [file Data_Sheet_1.ZIP › Raw date/Figure5/Figure5A/Input-Anti-Flag.jpg]

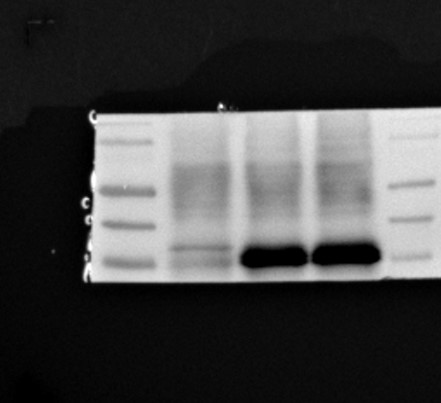

Supplement: Supplementary file 1 [file Data_Sheet_1.ZIP › Raw date/Figure5/Figure5C/Anti-Flag-Cell Lysate .jpg]

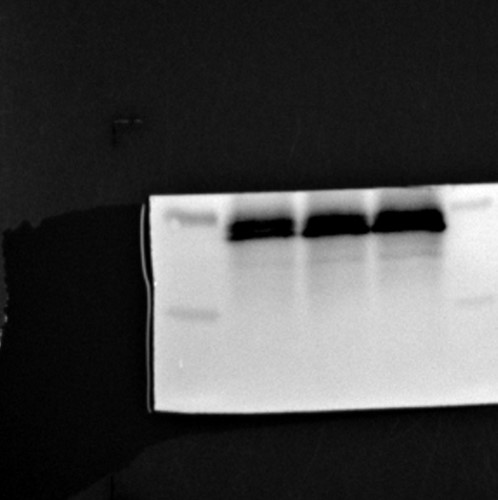

Supplement: Supplementary file 1 [file Data_Sheet_1.ZIP › Raw date/Figure5/Figure5C/Anti-Myc-Cell Lysate .jpg]

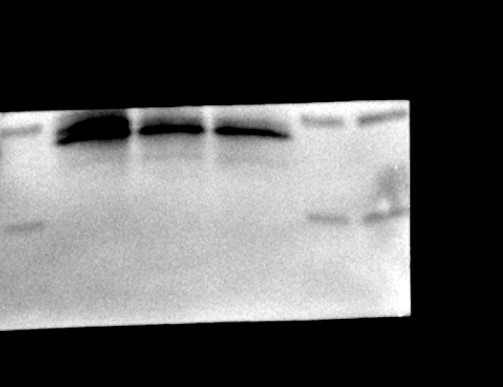

Supplement: Supplementary file 1 [file Data_Sheet_1.ZIP › Raw date/Figure5/Figure5C/Anti-Myc-VLP .jpg]

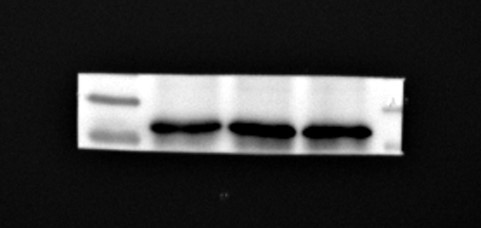

Supplement: Supplementary file 1 [file Data_Sheet_1.ZIP › Raw date/Figure5/Figure5C/GAPDH.jpg]

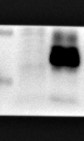

Supplement: Supplementary file 1 [file Data_Sheet_1.ZIP › Raw date/Supplementary Figure 1/Supplementary Figure1A/Anti-HA-IP.jpg]

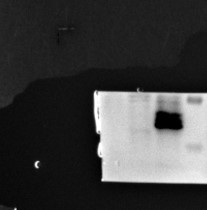

Supplement: Supplementary file 1 [file Data_Sheet_1.ZIP › Raw date/Supplementary Figure 1/Supplementary Figure1A/Anti-HA-input.jpg]

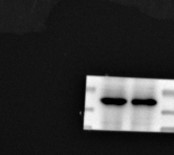

Supplement: Supplementary file 1 [file Data_Sheet_1.ZIP › Raw date/Supplementary Figure 1/Supplementary Figure1A/GAPDH.jpg]

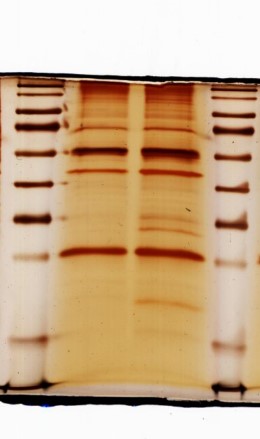

Supplement: Supplementary file 1 [file Data_Sheet_1.ZIP › Raw date/Supplementary Figure 1/Supplementary Figure1B/strip.jpg]

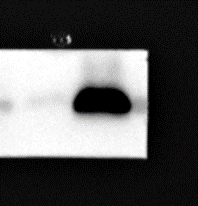

Supplement: Supplementary file 1 [file Data_Sheet_1.ZIP › Raw date/Supplementary Figure 2/Supplementary Figure 2A/M2-IP.jpg]

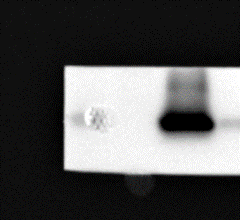

Supplement: Supplementary file 1 [file Data_Sheet_1.ZIP › Raw date/Supplementary Figure 2/Supplementary Figure 2A/M2-input.jpg]

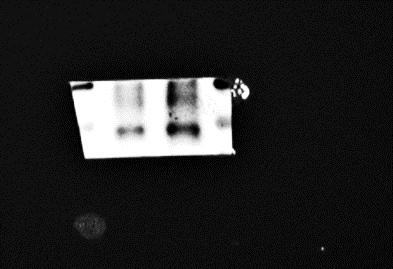

Supplement: Supplementary file 1 [file Data_Sheet_1.ZIP › Raw date/Supplementary Figure 2/Supplementary Figure 2A/YWHAG-IP.jpg]

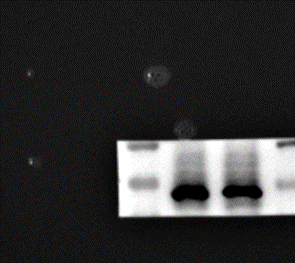

Supplement: Supplementary file 1 [file Data_Sheet_1.ZIP › Raw date/Supplementary Figure 2/Supplementary Figure 2A/YWHAG-input.jpg]

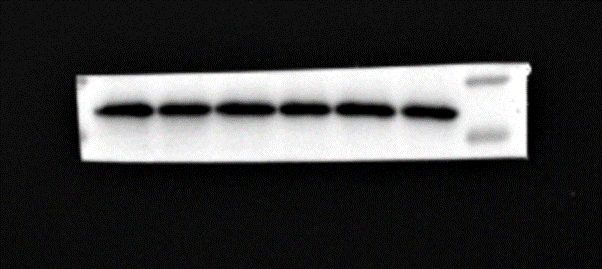

Supplement: Supplementary file 1 [file Data_Sheet_1.ZIP › Raw date/Supplementary Figure 3/Supplementary Figure 3A/1GAPDH.jpg]

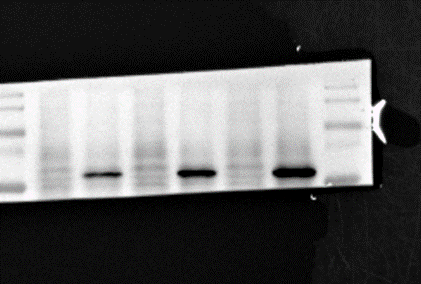

Supplement: Supplementary file 1 [file Data_Sheet_1.ZIP › Raw date/Supplementary Figure 3/Supplementary Figure 3A/Anti-NP.jpg]

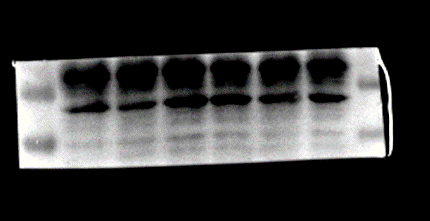

Supplement: Supplementary file 1 [file Data_Sheet_1.ZIP › Raw date/Supplementary Figure 3/Supplementary Figure 3A/Anti-YWHAG.jpg]

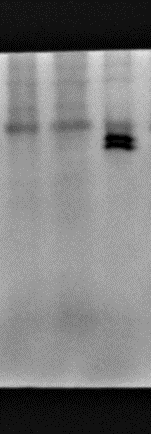

Supplement: Supplementary file 1 [file Data_Sheet_1.ZIP › Raw date/Supplementary Figure 4/Supplementary Figure 4A/Anti-Flag.jpg]

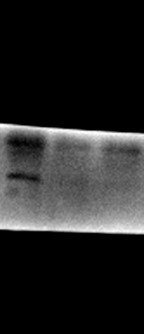

Supplement: Supplementary file 1 [file Data_Sheet_1.ZIP › Raw date/Supplementary Figure 4/Supplementary Figure 4A/Anti-YWHAG.jpg]

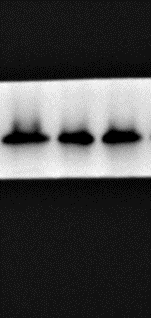

Supplement: Supplementary file 1 [file Data_Sheet_1.ZIP › Raw date/Supplementary Figure 4/Supplementary Figure 4A/GAPDH .jpg]

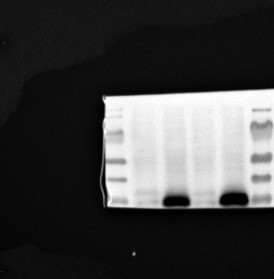

Supplement: Supplementary file 1 [file Data_Sheet_1.ZIP › Raw date/Supplementary Figure 5/Anti-Flag-input.jpg]

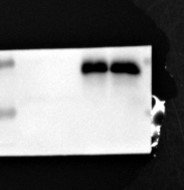

Supplement: Supplementary file 1 [file Data_Sheet_1.ZIP › Raw date/Supplementary Figure 5/Anti-Myc-IP.jpg]

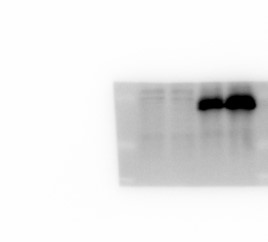

Supplement: Supplementary file 1 [file Data_Sheet_1.ZIP › Raw date/Supplementary Figure 5/Anti-Myc-input.jpg]

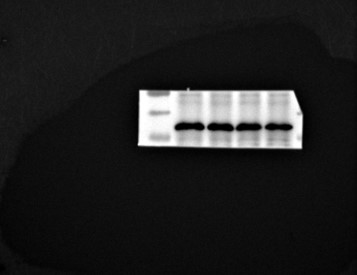

Supplement: Supplementary file 1 [file Data_Sheet_1.ZIP › Raw date/Supplementary Figure 5/GAPDH.jpg]

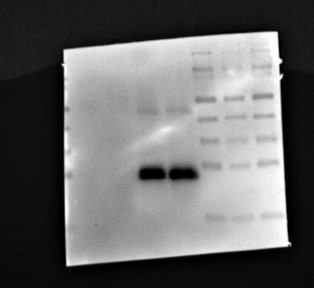

Supplement: Supplementary file 1 [file Data_Sheet_1.ZIP › Raw date/Supplementary Figure 5/P-(Ser+Try)-M2-IP.jpg]
