# Supplementary material for: YWHAG inhibits influenza a virus replication by suppressing the release of viral M2 protein
Source: Front Microbiol. 2022 Jul 19;13:951009. doi: 10.3389/fmicb.2022.951009 (PMC9343881; doi:10.3389/fmicb.2022.951009)
Supplement: Supplementary file 2 [file Data_Sheet_2.DOCX]

Supplementary Material

# Supplementary Figures and Tables

## Supplementary Figures


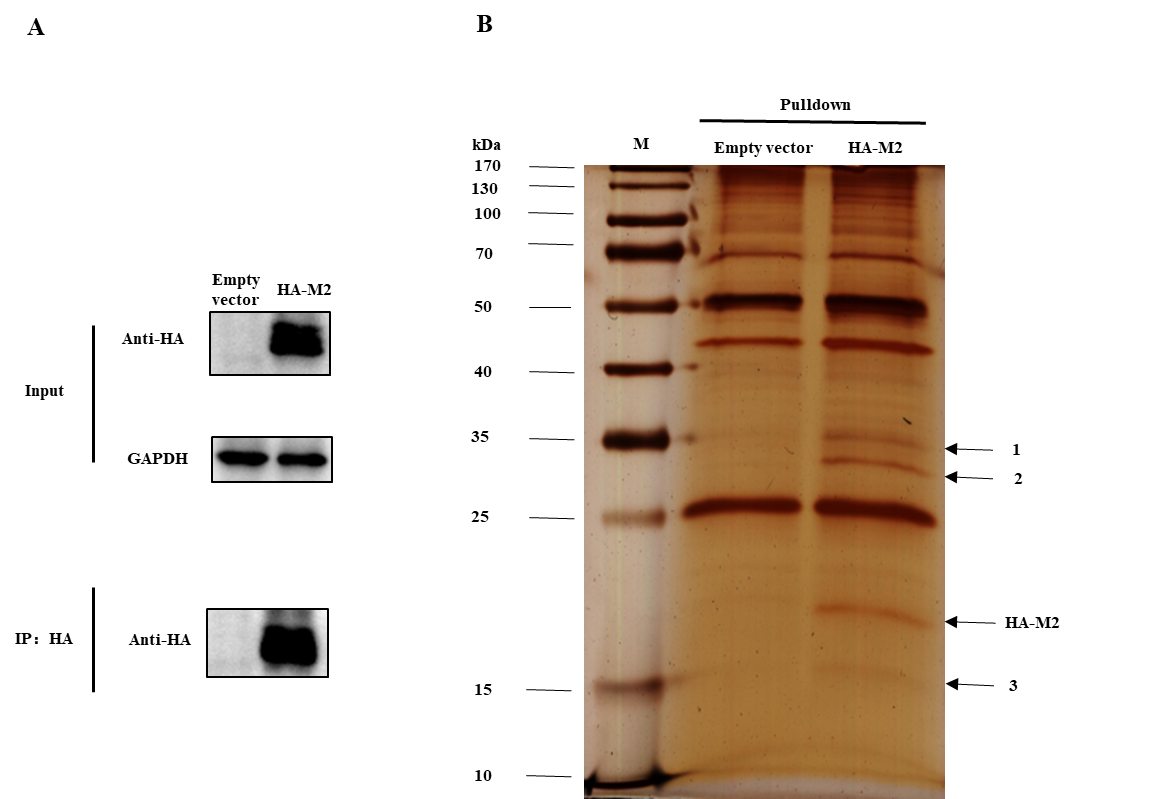


**Supplementary Figure 1.** Identification of influenza virus M2-associated factors by immunoprecipitation-mass spectrometry (IP/MS). **(A and B)** Immunoprecipitates of anti-HA beads from HA-M2 overexpressed HEK293T cells were analyzed by Western blotting **(A)** and silver staining. The differential strips(1.2.3) were cut down for mass spectrometry analysis.


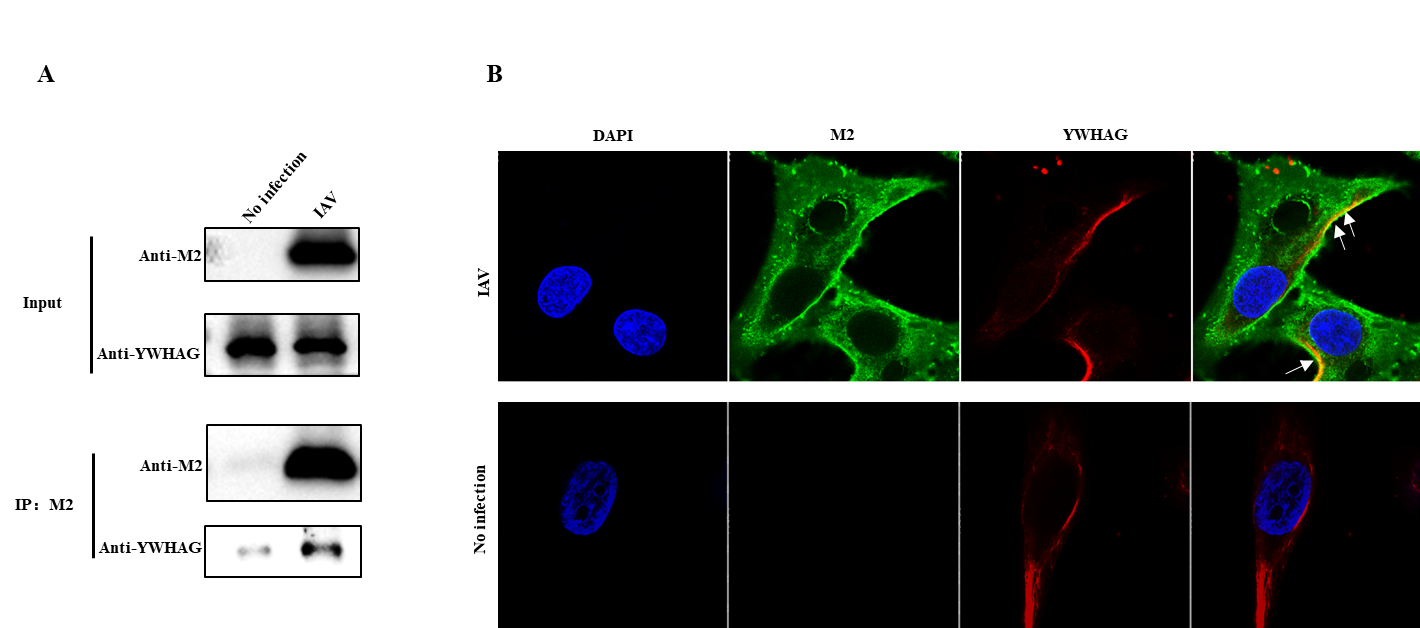


**Supplementary Figure 2.** IAV M2 interacts with endogenous YWHAG. **(A and B)** PR8 infected wide-type A549 cells for 12h, **(A)** cell lysates were incubated with anti-M2 protein A/G agarous for 2 h at 4 °C, and IP eluates were subjected to western blotting analysis. **(B)** cells were stained with the anti-M2 mouse green fluorescent antibody and anti-YWHAG ribbit red fluorescent antibody, the nuclei was stained by DAPI.


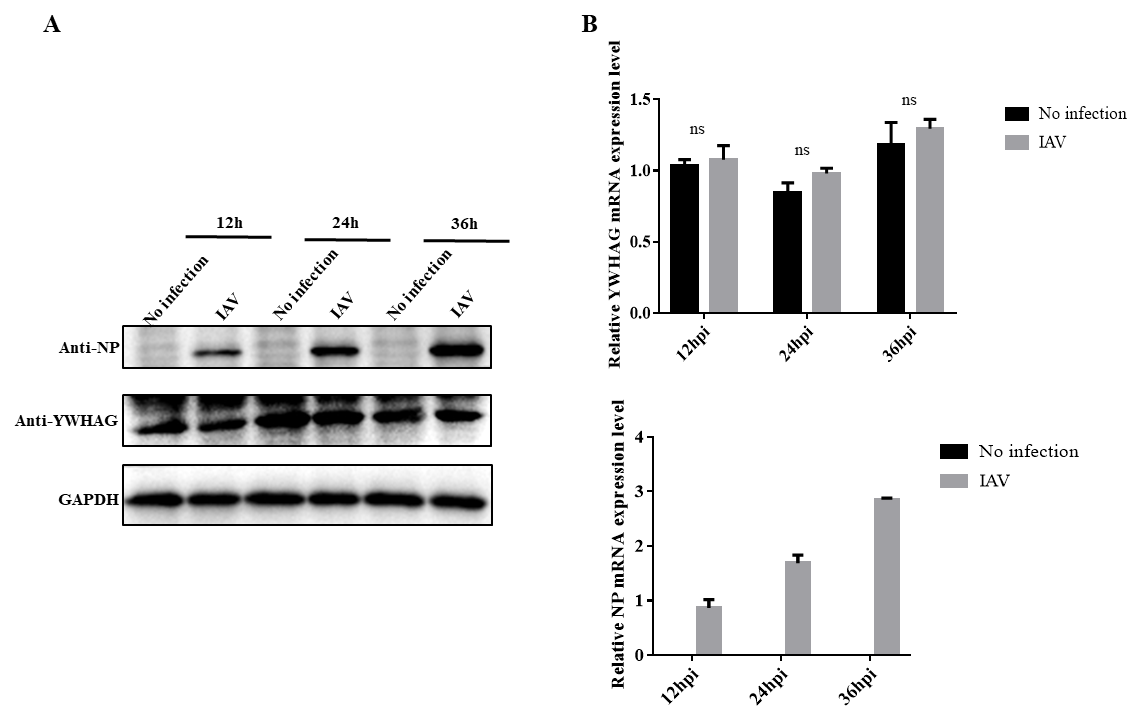


**Supplementary Figure 3.** IAV does not change the expression of YWHAG. **(A and B)** Wide-type A549 cells were cultured in 12-well plates and treated with PR8 at MOI=0.01 or no infection, respectively. Cells were collected at 12h, 24h and 36h to detect YWHAG expression by western blotting **(A)** and RT-qPCR **(B)**.

**
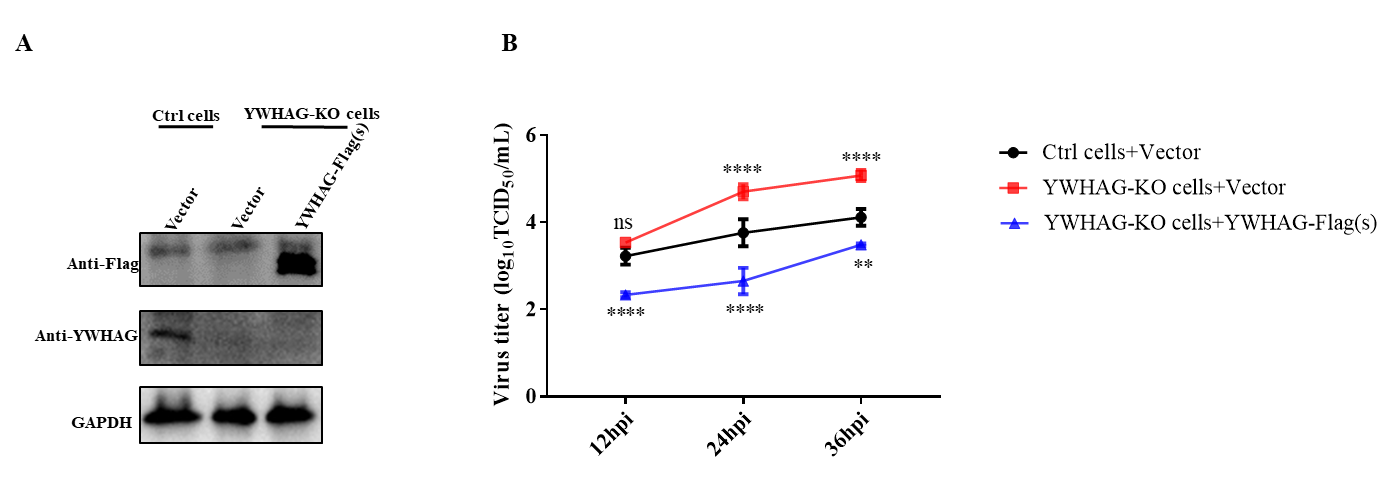
**

**Supplementary Figure 4.** Validation of YWHAG knockout in YWHAG-KO cells. **(A)** Flag-tagged YWHAG(s) and empty vector transfected into YWHAG-KO or control cells for 24h, cells were collected and lysed for western blotting. **(B)** YWHAG-KO and control cells were transfected with reconstituted Flag-tagged YWHAG (s) and empty vector and following infected with PR8 H1N1 virus (MOI= 0.01). At 12-36 hpi, we collected the supernatants for TCID_50_ assay.


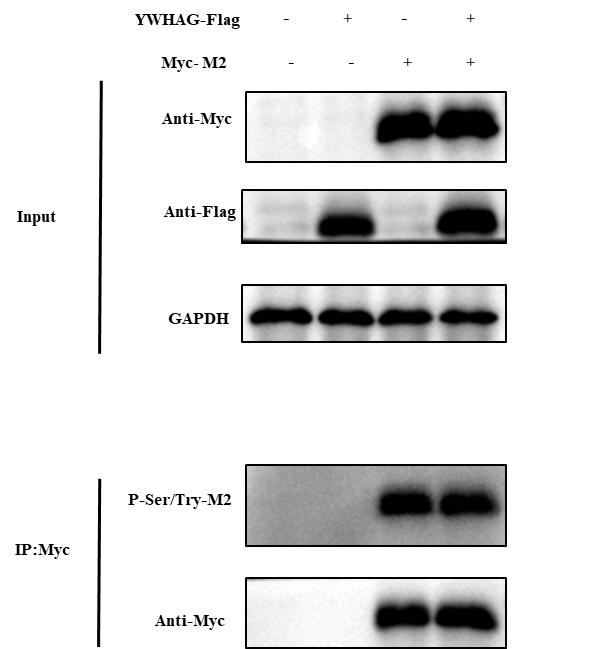


**Supplementary Figuer 5.** YWHAG dose not affect the phosphorylation of virul M2. Flag-tagged YWHAG or empty vector co-transfected with Myc-tagged M2 or empty vector in HEK293T cells. After 24h, cell lysates were collected and immunoprecipitated with anti-Myc beads, followed by western blotting with a rabbit anti-Myc pAb, a rabbit anti- p-Ser/Tyr mAb to detect the level of total M2, serine/ tyrosine -phosphorylated M2, respectively.

## Supplementary Table

Table 1. One hundred and fifteen host proteins that interacted with M2 were identified by LC-MS/MS mass spectrometry.

| Accession | Gene | Description | Mw(kDa ) |
| --- | --- | --- | --- |
| Q14653 | IRF3 | Interferon regulatory factor 3 OS=Homo sapiens OX=9606 GN=IRF3 PE=1 SV=1 | 47.219 |
| P35579 | MYH9 | Myosin-9 OS=Homo sapiens OX=9606 GN=MYH9 PE=1 SV=4 | 226.532 |
| P04259 | KRT6B | Keratin, type II cytoskeletal 6B OS=Homo sapiens OX=9606 GN=KRT6B PE=1 SV=5 | 60.067 |
| P63261 | ACTG1 | Actin, cytoplasmic 2 OS=Homo sapiens OX=9606 GN=ACTG1 PE=1 SV=1 | 41.793 |
| P09651 | HNRNPA1 | Heterogeneous nuclear ribonucleoprotein A1 OS=Homo sapiens OX=9606 GN=HNRNPA1 PE=1 SV=5 | 38.747 |
| P05141 | SLC25A5 | ADP/ATP translocase 2 OS=Homo sapiens OX=9606 GN=SLC25A5 PE=1 SV=7 | 32.852 |
| P08670 | VIM | Vimentin OS=Homo sapiens OX=9606 GN=VIM PE=1 SV=4 | 53.652 |
| P31942 | HNRNPH3 | Heterogeneous nuclear ribonucleoprotein H3 OS=Homo sapiens OX=9606 GN=HNRNPH3 PE=1 SV=2 | 36.926 |
| P50402 | EMD | Emerin OS=Homo sapiens OX=9606 GN=EMD PE=1 SV=1 | 28.994 |
| Q07666 | KHDRBS1 | KH domain-containing, RNA-binding, signal transduction-associated protein 1 OS=Homo sapiens OX=9606 GN=KHDRBS1 PE=1 SV=1 | 48.227 |
| P62633 | CNBP | CCHC-type zinc finger nucleic acid binding protein OS=Homo sapiens OX=9606 GN=CNBP PE=1 SV=1 | 19.463 |
| P62750 | RPL23A | 60S ribosomal protein L23a OS=Homo sapiens OX=9606 GN=RPL23A PE=1 SV=1 | 17.695 |
| P62917 | RPL8 | 60S ribosomal protein L8 OS=Homo sapiens OX=9606 GN=RPL8 PE=1 SV=2 | 28.025 |
| P23246 | SFPQ | Splicing factor, proline- and glutamine-rich OS=Homo sapiens OX=9606 GN=SFPQ PE=1 SV=2 | 76.149 |
| P62280 | RPS11 | 40S ribosomal protein S11 OS=Homo sapiens OX=9606 GN=RPS11 PE=1 SV=3 | 18.431 |
| Q96C19 | EFHD2 | EF-hand domain-containing protein D2 OS=Homo sapiens OX=9606 GN=EFHD2 PE=1 SV=1 | 26.697 |
| Q99700 | ATXN2 | Ataxin-2 OS=Homo sapiens OX=9606 GN=ATXN2 PE=1 SV=2 | 140.283 |
| Q9Y5M8 | SRPRB | Signal recognition particle receptor subunit beta OS=Homo sapiens OX=9606 GN=SRPRB PE=1 SV=3 | 29.702 |
| O00571 | DDX3X | ATP-dependent RNA helicase DDX3X OS=Homo sapiens OX=9606 GN=DDX3X PE=1 SV=3 | 73.243 |
| O15027 | SEC16A | Protein transport protein Sec16A OS=Homo sapiens OX=9606 GN=SEC16A PE=1 SV=4 | 251.894 |
| P07910 | HNRNPC | Heterogeneous nuclear ribonucleoproteins C1/C2 OS=Homo sapiens OX=9606 GN=HNRNPC PE=1 SV=4 | 33.67 |
| P12236 | SLC25A6 | ADP/ATP translocase 3 OS=Homo sapiens OX=9606 GN=SLC25A6 PE=1 SV=4 | 32.866 |
| P18124 | RPL7 | 60S ribosomal protein L7 OS=Homo sapiens OX=9606 GN=RPL7 PE=1 SV=1 | 29.226 |
| P18621 | RPL17 | 60S ribosomal protein L17 OS=Homo sapiens OX=9606 GN=RPL17 PE=1 SV=3 | 21.397 |
| P35580 | MYH10 | Myosin-10 OS=Homo sapiens OX=9606 GN=MYH10 PE=1 SV=3 | 228.999 |
| P37802 | TAGLN2 | Transgelin-2 OS=Homo sapiens OX=9606 GN=TAGLN2 PE=1 SV=3 | 22.391 |
| P38159 | RBMX | RNA-binding motif protein, X chromosome OS=Homo sapiens OX=9606 GN=RBMX PE=1 SV=3 | 42.332 |
| P43243 | MATR3 | Matrin-3 OS=Homo sapiens OX=9606 GN=MATR3 PE=1 SV=2 | 94.623 |
| P51991 | HNRNPA3 | Heterogeneous nuclear ribonucleoprotein A3 OS=Homo sapiens OX=9606 GN=HNRNPA3 PE=1 SV=2 | 39.595 |
| P61981 | YWHAG | 14-3-3 pro tein gamma OS=Homo sapiens OX=9606 GN=YWHAG PE=1 SV=2 | 28.303 |
| P62263 | RPS14 | 40S ribosomal protein S14 OS=Homo sapiens OX=9606 GN=RPS14 PE=1 SV=3 | 16.273 |
| P62899 | RPL31 | 60S ribosomal protein L31 OS=Homo sapiens OX=9606 GN=RPL31 PE=1 SV=1 | 14.463 |
| Q02978 | SLC25A11 | Mitochondrial 2-oxoglutarate/malate carrier protein OS=Homo sapiens OX=9606 GN=SLC25A11 PE=1 SV=3 | 34.062 |
| Q13151 | HNRNPA0 | Heterogeneous nuclear ribonucleoprotein A0 OS=Homo sapiens OX=9606 GN=HNRNPA0 PE=1 SV=1 | 30.841 |
| Q15365 | PCBP1 | Poly(rC)-binding protein 1 OS=Homo sapiens OX=9606 GN=PCBP1 PE=1 SV=2 | 37.498 |
| Q15637 | SF1 | Splicing factor 1 OS=Homo sapiens OX=9606 GN=SF1 PE=1 SV=4 | 68.33 |
| Q5T2N8 | ATAD3C | ATPase family AAA domain-containing protein 3C OS=Homo sapiens OX=9606 GN=ATAD3C PE=2 SV=2 | 46.38 |
| Q8TAP9 | MPLKIP | M-phase-specific PLK1-interacting protein OS=Homo sapiens OX=9606 GN=MPLKIP PE=1 SV=1 | 19.147 |
| Q8WWM7 | ATXN2L | Ataxin-2-like protein OS=Homo sapiens OX=9606 GN=ATXN2L PE=1 SV=2 | 113.374 |
| Q92733 | PRCC | Proline-rich protein PRCC OS=Homo sapiens OX=9606 GN=PRCC PE=1 SV=1 | 52.418 |
| Q92804 | TAF15 | TATA-binding protein-associated factor 2N OS=Homo sapiens OX=9606 GN=TAF15 PE=1 SV=1 | 61.83 |
| Q92841 | DDX17 | Probable ATP-dependent RNA helicase DDX17 OS=Homo sapiens OX=9606 GN=DDX17 PE=1 SV=2 | 80.272 |
| Q9BQE3 | TUBA1C | Tubulin alpha-1C chain OS=Homo sapiens OX=9606 GN=TUBA1C PE=1 SV=1 | 49.895 |
| Q9H361 | PABPC3 | Polyadenylate-binding protein 3 OS=Homo sapiens OX=9606 GN=PABPC3 PE=1 SV=2 | 70.031 |
| Q9H3N1 | TMX1 | Thioredoxin-related transmembrane protein 1 OS=Homo sapiens OX=9606 GN=TMX1 PE=1 SV=1 | 31.791 |
| O14979 | HNRNPDL | Heterogeneous nuclear ribonucleoprotein D-like OS=Homo sapiens OX=9606 GN=HNRNPDL PE=1 SV=3 | 46.438 |
| O15511 | ARPC5 | Actin-related protein 2/3 complex subunit 5 OS=Homo sapiens OX=9606 GN=ARPC5 PE=1 SV=3 | 16.32 |
| O60506 | SYNCRIP | Heterogeneous nuclear ribonucleoprotein Q OS=Homo sapiens OX=9606 GN=SYNCRIP PE=1 SV=2 | 69.603 |
| O60762 | DPM1 | Dolichol-phosphate mannosyltransferase subunit 1 OS=Homo sapiens OX=9606 GN=DPM1 PE=1 SV=1 | 29.634 |
| O75934 | BCAS2 | Pre-mRNA-splicing factor SPF27 OS=Homo sapiens OX=9606 GN=BCAS2 PE=1 SV=1 | 26.131 |
| P05089 | ARG1 | Arginase-1 OS=Homo sapiens OX=9606 GN=ARG1 PE=1 SV=2 | 34.735 |
| P06753 | TPM3 | Tropomyosin alpha-3 chain OS=Homo sapiens OX=9606 GN=TPM3 PE=1 SV=2 | 32.95 |
| P12235 | SLC25A4 | ADP/ATP translocase 1 OS=Homo sapiens OX=9606 GN=SLC25A4 PE=1 SV=4 | 33.065 |
| P17844 | DDX5 | Probable ATP-dependent RNA helicase DDX5 OS=Homo sapiens OX=9606 GN=DDX5 PE=1 SV=1 | 69.148 |
| P20719 | HOXA5 | Homeobox protein Hox-A5 OS=Homo sapiens OX=9606 GN=HOXA5 PE=1 SV=2 | 29.345 |
| P22626 | HNRNPA2B1 | Heterogeneous nuclear ribonucleoproteins A2/B1 OS=Homo sapiens OX=9606 GN=HNRNPA2B1 PE=1 SV=2 | 37.43 |
| P26373 | RPL13 | 60S ribosomal protein L13 OS=Homo sapiens OX=9606 GN=RPL13 PE=1 SV=4 | 24.261 |
| P26599 | PTBP1 | Polypyrimidine tract-binding protein 1 OS=Homo sapiens OX=9606 GN=PTBP1 PE=1 SV=1 | 57.221 |
| P30050 | RPL12 | 60S ribosomal protein L12 OS=Homo sapiens OX=9606 GN=RPL12 PE=1 SV=1 | 17.819 |
| P38646 | HSPA9 | Stress-70 protein, mitochondrial OS=Homo sapiens OX=9606 GN=HSPA9 PE=1 SV=2 | 73.681 |
| P52597 | HNRNPF | Heterogeneous nuclear ribonucleoprotein F OS=Homo sapiens OX=9606 GN=HNRNPF PE=1 SV=3 | 45.672 |
| P52907 | CAPZA1 | F-actin-capping protein subunit alpha-1 OS=Homo sapiens OX=9606 GN=CAPZA1 PE=1 SV=3 | 32.923 |
| P60660 | MYL6 | Myosin light polypeptide 6 OS=Homo sapiens OX=9606 GN=MYL6 PE=1 SV=2 | 16.93 |
| P61247 | RPS3A | 40S ribosomal protein S3a OS=Homo sapiens OX=9606 GN=RPS3A PE=1 SV=2 | 29.945 |
| P61978 | HNRNPK | Heterogeneous nuclear ribonucleoprotein K OS=Homo sapiens OX=9606 GN=HNRNPK PE=1 SV=1 | 50.976 |
| P62851 | RPS25 | 40S ribosomal protein S25 OS=Homo sapiens OX=9606 GN=RPS25 PE=1 SV=1 | 13.742 |
| P81605 | DCD | Dermcidin OS=Homo sapiens OX=9606 GN=DCD PE=1 SV=2 | 11.284 |
| P84098 | RPL19 | 60S ribosomal protein L19 OS=Homo sapiens OX=9606 GN=RPL19 PE=1 SV=1 | 23.466 |
| Q04917 | YWHAH | 14-3-3 protein eta OS=Homo sapiens OX=9606 GN=YWHAH PE=1 SV=4 | 28.219 |
| Q14247 | CTTN | Src substrate cortactin OS=Homo sapiens OX=9606 GN=CTTN PE=1 SV=2 | 61.586 |
| Q15233 | NONO | Non-POU domain-containing octamer-binding protein OS=Homo sapiens OX=9606 GN=NONO PE=1 SV=4 | 54.232 |
| Q15717 | ELAVL1 | ELAV-like protein 1 OS=Homo sapiens OX=9606 GN=ELAVL1 PE=1 SV=2 | 36.092 |
| Q3MHD2 | LSM12 | Protein LSM12 homolog OS=Homo sapiens OX=9606 GN=LSM12 PE=1 SV=2 | 21.701 |
| Q5VTE0 | EEF1A1P5 | Putative elongation factor 1-alpha-like 3 OS=Homo sapiens OX=9606 GN=EEF1A1P5 PE=5 SV=1 | 50.185 |
| Q6NZY4 | ZCCHC8 | Zinc finger CCHC domain-containing protein 8 OS=Homo sapiens OX=9606 GN=ZCCHC8 PE=1 SV=2 | 78.577 |
| Q7RTV0 | PHF5A | PHD finger-like domain-containing protein 5A OS=Homo sapiens OX=9606 GN=PHF5A PE=1 SV=1 | 12.405 |
| Q8IWS0 | PHF6 | PHD finger protein 6 OS=Homo sapiens OX=9606 GN=PHF6 PE=1 SV=1 | 41.29 |
| Q8WZA9 | IRGQ | Immunity-related GTPase family Q protein OS=Homo sapiens OX=9606 GN=IRGQ PE=1 SV=1 | 62.717 |
| Q92567 | FAM168A | Protein FAM168A OS=Homo sapiens OX=9606 GN=FAM168A PE=1 SV=2 | 26.184 |
| Q9BPX5 | ARPC5L | Actin-related protein 2/3 complex subunit 5-like protein OS=Homo sapiens OX=9606 GN=ARPC5L PE=1 SV=1 | 16.941 |
| Q9BUJ2 | HNRNPUL1 | Heterogeneous nuclear ribonucleoprotein U-like protein 1 OS=Homo sapiens OX=9606 GN=HNRNPUL1 PE=1 SV=2 | 95.739 |
| Q9HAV0 | GNB4 | Guanine nucleotide-binding protein subunit beta-4 OS=Homo sapiens OX=9606 GN=GNB4 PE=1 SV=3 | 37.567 |
| Q9NQX3 | GPHN | Gephyrin OS=Homo sapiens OX=9606 GN=GPHN PE=1 SV=1 | 79.748 |
| Q9NR30 | DDX21 | Nucleolar RNA helicase 2 OS=Homo sapiens OX=9606 GN=DDX21 PE=1 SV=5 | 87.344 |
| Q9P258 | RCC2 | Protein RCC2 OS=Homo sapiens OX=9606 GN=RCC2 PE=1 SV=2 | 56.085 |
| Q9UGP4 | LIMD1 | LIM domain-containing protein 1 OS=Homo sapiens OX=9606 GN=LIMD1 PE=1 SV=1 | 72.19 |
| Q9Y2W1 | THRAP3 | Thyroid hormone receptor-associated protein 3 OS=Homo sapiens OX=9606 GN=THRAP3 PE=1 SV=2 | 108.666 |
| Q9Y3I0 | RTCB | RNA-splicing ligase RtcB homolog OS=Homo sapiens OX=9606 GN=RTCB PE=1 SV=1 | 55.21 |
| Q9Y3R5 | DOP1B | Protein dopey-2 OS=Homo sapiens OX=9606 GN=DOP1B PE=1 SV=5 | 258.23 |
| Q9Y3Y2 | CHTOP | Chromatin target of PRMT1 protein OS=Homo sapiens OX=9606 GN=CHTOP PE=1 SV=2 | 26.397 |
| A0A075B6R9 | IGKV2D-24 | Probable non-functional immunoglobulin kappa variable 2D-24 OS=Homo sapiens OX=9606 GN=IGKV2D-24 PE=1 SV=1 | 13.079 |
| A6NJB7 | PRR19 | Proline-rich protein 19 OS=Homo sapiens OX=9606 GN=PRR19 PE=1 SV=1 | 38.716 |
| O14523 | C2CD2L | Phospholipid transfer protein C2CD2L OS=Homo sapiens OX=9606 GN=C2CD2L PE=1 SV=3 | 76.181 |
| O95932 | TGM6 | Protein-glutamine gamma-glutamyltransferase 6 OS=Homo sapiens OX=9606 GN=TGM6 PE=1 SV=3 | 79.312 |
| P06733 | ENO1 | Alpha-enolase OS=Homo sapiens OX=9606 GN=ENO1 PE=1 SV=2 | 47.169 |
| P19105 | MYL12A | Myosin regulatory light chain 12A OS=Homo sapiens OX=9606 GN=MYL12A PE=1 SV=2 | 19.794 |
| P36578 | RPL4 | 60S ribosomal protein L4 OS=Homo sapiens OX=9606 GN=RPL4 PE=1 SV=5 | 47.697 |
| P52849 | NDST2 | Bifunctional heparan sulfate N-deacetylase/N-sulfotransferase 2 OS=Homo sapiens OX=9606 GN=NDST2 PE=1 SV=1 | 100.875 |
| P60709 | ACTB | Actin, cytoplasmic 1 OS=Homo sapiens OX=9606 GN=ACTB PE=1 SV=1 | 41.737 |
| Q00839 | HNRNPU | Heterogeneous nuclear ribonucleoprotein U OS=Homo sapiens OX=9606 GN=HNRNPU PE=1 SV=6 | 90.584 |
| Q13868 | EXOSC2 | Exosome complex component RRP4 OS=Homo sapiens OX=9606 GN=EXOSC2 PE=1 SV=2 | 32.789 |
| Q16643 | DBN1 | Drebrin OS=Homo sapiens OX=9606 GN=DBN1 PE=1 SV=4 | 71.429 |
| Q6T4R5 | NHS | Nance-Horan syndrome protein OS=Homo sapiens OX=9606 GN=NHS PE=1 SV=2 | 179.135 |
| Q8N9B5 | JMY | Junction-mediating and -regulatory protein OS=Homo sapiens OX=9606 GN=JMY PE=1 SV=2 | 111.445 |
| Q8ND83 | SLAIN1 | SLAIN motif-containing protein 1 OS=Homo sapiens OX=9606 GN=SLAIN1 PE=1 SV=3 | 60.595 |
| Q8NG31 | KNL1 | Kinetochore scaffold 1 OS=Homo sapiens OX=9606 GN=KNL1 PE=1 SV=3 | 265.391 |
| Q96PK6 | RBM14 | RNA-binding protein 14 OS=Homo sapiens OX=9606 GN=RBM14 PE=1 SV=2 | 69.492 |
| Q99459 | CDC5L | Cell division cycle 5-like protein OS=Homo sapiens OX=9606 GN=CDC5L PE=1 SV=2 | 92.251 |
| Q9BZJ0 | CRNKL1 | Crooked neck-like protein 1 OS=Homo sapiens OX=9606 GN=CRNKL1 PE=1 SV=4 | 100.452 |
| Q9C091 | GREB1L | GREB1-like protein OS=Homo sapiens OX=9606 GN=GREB1L PE=1 SV=2 | 214.354 |
| Q9H2K2 | TNKS2 | Poly [ADP-ribose] polymerase tankyrase-2 OS=Homo sapiens OX=9606 GN=TNKS2 PE=1 SV=1 | 126.918 |
| Q9H607 | OCEL1 | Occludin/ELL domain-containing protein 1 OS=Homo sapiens OX=9606 GN=OCEL1 PE=2 SV=1 | 29.404 |
| Q9NP81 | SARS2 | Serine--tRNA ligase, mitochondrial OS=Homo sapiens OX=9606 GN=SARS2 PE=1 SV=1 | 58.283 |
| Q9UMS4 | PRPF19 | Pre-mRNA-processing factor 19 OS=Homo sapiens OX=9606 GN=PRPF19 PE=1 SV=1 | 55.181 |
| Q9Y4D8 | HECTD4 | Probable E3 ubiquitin-protein ligase HECTD4 OS=Homo sapiens OX=9606 GN=HECTD4 PE=1 SV=5 | 439.344 |
